# The Origin and Application of Cardiomyocyte-Derived Small Extracellular Vesicles: A Systematic Review

**DOI:** 10.7150/ijms.133199

**Published:** 2026-05-18

**Authors:** Shaojiao Liu, Yu Teng, Sha Su, Meng Wang, Lei Wang, Mingjing Zhao

**Affiliations:** 1 Key Laboratory of Chinese Internal Medicine of Ministry of Education, Dongzhimen Hospital, Beijing University of Chinese Medicine, Beijing, 100700, China.; 2 Institute of Cardiovascular Diseases, Beijing University of Chinese Medicine, Beijing, 100700, China.

**Keywords:** cardiomyocytes, small extracellular vesicles, cardiovascular diseases, intercellular communication, biomarkers

## Abstract

**Background/Aims**: Cardiomyocyte-derived small extracellular vesicles (CM-sEVs) have emerged as important mediators of intercellular communication in cardiovascular diseases (CVDs). However, their origin-tracing markers, molecular signatures, and clinical applications remain incompletely characterized and lack systematic synthesis. This systematic review aimed to comprehensively evaluate CM-sEVs-specific markers, disease-associated cargos alterations, and their roles in intercellular communication.

**Methods:**

A PRISMA-guided systematic search was conducted across major databases, including Web of Science, PubMed, Embase, and the Cochrane Library. Study screening, data extraction, and quality assessment were independently performed by two investigators according to predefined eligibility criteria.

**Results:**

Thirty-four studies were included and three sets of information were systematically analyzed. Ldb3, Ambra1, and CD172a were verified as potential origin-tracing markers of CM-sEVs, and miR-208a, cTnT/Tnnt2, and α-MHC/Myh6 served as auxiliary markers. Several CM-sEVs-associated molecules, including CD172a, Ambra1, miR-9-5p, and lncRNA HCG15, demonstrated diagnostic or prognostic potential in CVDs populations. Functionally, CM-sEVs regulate fibrosis, angiogenesis, autophagy, and immune responses through cardiomyocyte-noncardiomyocyte communication networks.

**Conclusion:**

This review systematically summarizes current evidence on potential origin-tracing markers, cargos characteristics, and intercellular communication roles of CM-sEVs, providing a theoretical basis for their identification and translational application in cardiovascular diseases.

## 1. Introduction

Small extracellular vesicles (sEVs) are a subpopulation of extracellular vesicles (EVs), typically defined as lipid bilayer-enclosed vesicles with a diameter of less than 200 nm. They are actively secreted by cells and can carry a variety of biomolecules, including proteins, lipids, and nucleic acids 1. Since the sEVs circulate stably in body fluids and reflect the pathophysiological status of their parent cells, they are considered as potential biomarkers for disease diagnosis. They are also involved in regulating target cell activities by transferring functional molecules between cells 2,3. Several studies in recent years have demonstrated that sEVs play crucial roles in the onset and progression of cardiovascular diseases (CVDs) 4. Their abundance and molecular cargos undergo dynamic changes in response to the physiological or pathological states of the parent cells. Therefore, sEVs participate in key pathological processes such as myocardial fibrosis 5, autophagy 6, immune regulation 7, and angiogenesis 8. Therefore, sEVs are promising diagnostic tools and therapeutic targets for CVDs. Among sEVs originating from different cell types, cardiomyocyte-derived sEVs (CM-sEVs) have attracted significant attention from the research and clinical community because cardiomyocytes constitute the major functional cell type of the heart and directly determine its contractile and pumping function 9. During pathological conditions, cardiomyocytes undergo intrinsic functional impairment and secrete sEVs enriched with specific proteins and RNAs that modulate the behavior of neighboring and distant cells 10,11. Therefore, CM-sEVs act as key mediators in cardiac remodeling, heart failure (HF), and the progression of other CVDs.

The *in vivo* tracing of CM-sEVs is challenging because of the heterogeneous population of sEVs derived from diverse cell types. Currently, accurate *in vivo* identification of CM-sEVs faces several obstacles. First, there is still a lack of widely accepted origin-tracing markers to distinguish vesicles from different cell types *in vivo*, which hampers the accurate identification of CM-sEVs. Second, the current evidence on CM-sEVs is mostly obtained from *in vitro* studies, and systematic investigations regarding their *in vivo* dynamics and functional roles in CVDs are still lacking. This review focuses on three core objectives. First, we summarize the origin-tracing markers of CM-sEVs and their screening methods and applications to identify the most representative molecular signatures for their *in vivo* identification and isolation. Second, to delineate the roles and alterations of CM-sEVs across a spectrum of CVDs, including ischemic heart disease, HF, valvular disorders, and arrhythmias. Third, to elucidate the mechanisms underlying CM-sEVs-mediated intercellular communication both *in vivo* and *in vitro*, with greater emphasis on their interactions with cardiomyocytes, fibroblasts, endothelial cells, and immune cells. By synthesizing these aspects, this review aims to clarify the characteristic molecular profiles of CM-sEVs, assess their potential as diagnostic biomarkers, and advance our understanding of the mechanisms by which CM-sEVs mediate intercellular communication in the cardiovascular system.

## 2. Methods

This systematic review was based on the PRISMA 2020 Statement 12.

### 2.1 Data Sources and Search Strategy

We searched Web of Science, PubMed, Embase, and the Cochrane Library for studies published up to October 2025, using the keywords “Heart,” “Myocardium,” “Myocytes, cardiac,” “Extracellular vesicles,” “Exosomes,” and related terms. The full search strategy is provided in the Supplementary Materials (Table S1). To ensure comprehensive coverage, no filters or restrictions were applied during the search. In addition, the reference lists of all identified studies and relevant review articles were manually screened to identify additional pertinent publications.

### 2.2 Inclusion and Exclusion Criteria

#### 2.2.1 Inclusion criteria

Studies focusing on sEVs derived from cardiomyocytes and confirmed at least by cardiomyocyte culture; studies in which changes in the abundance or cargos of sEVs were validated *in vivo* in patients or animal models.

#### 2.2.2 Exclusion criteria

Articles with incomplete information, review papers, conference abstracts, or duplicate publications.

### 2.3 Data Extraction

All references were managed and deduplicated using EndNote X9. Two methodologically trained reviewers independently screened the studies in three stages: (1) titles were first screened to exclude non-original articles and studies irrelevant to the research topic; (2) abstracts were then reviewed to further remove studies not meeting the inclusion criteria; and (3) the full texts of the remaining articles were examined to determine final eligibility. Any discrepancies between the two reviewers were resolved through discussion, and a third reviewer was consulted when necessary. For each included study, the following information was extracted: title, publication year, authors, journal, country/region, and study species. For clinical studies, data were collected on study design, disease type, sex, age, sample size, and group allocation. For animal experiments, details on species, sex, age, or body weight, grouping, model type, and modeling methods were recorded. For cellular experiments, information regarding cell types and treatment conditions was documented. In addition, data on CM-sEVs characterization methods, alterations in their quantity and molecular cargos, recipient cell types, target molecules, and related mechanisms were extracted. All extracted data were cross-checked and verified for accuracy and completeness.

### 2.4 Quality Assessment

The quality of cross-sectional studies was assessed according to the Agency for Healthcare Research and Quality criteria 13. Longitudinal cohort studies were evaluated using the Newcastle-Ottawa Scale (NOS) 14. The SYRCLE's risk of bias tool was used to assess the quality of animal studies 15.

## 3. Results

### 3.1 Results of Literature Screening

**Figure 1:**
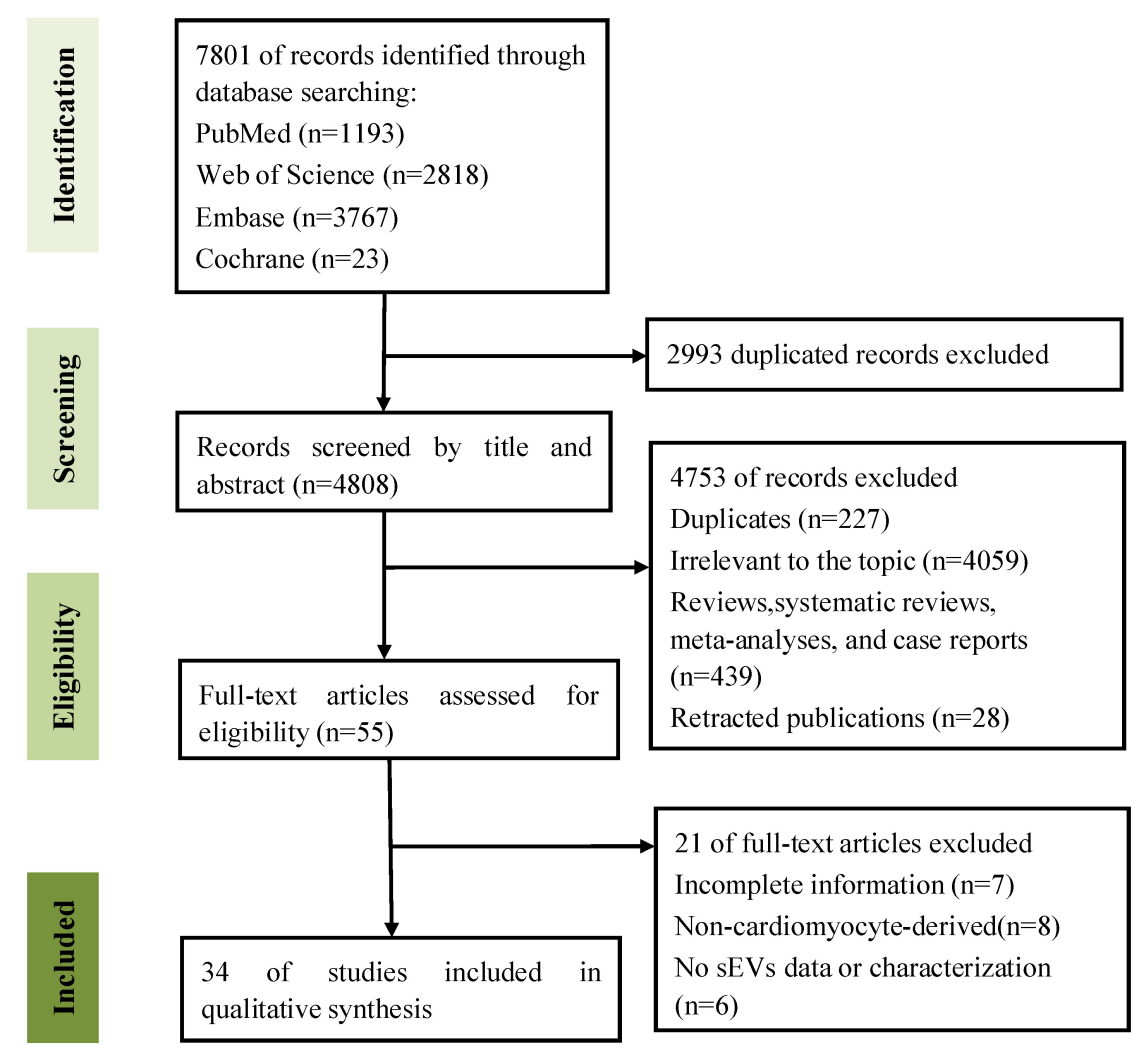
Flow chart of literature screening and selection.

The literature selection process is shown in Figure 1. We retrieved 7,801 records from the PubMed, Web of Science, Embase, and Cochrane Library databases. After removing duplicates, 4,808 records were screened based on titles and abstracts. Subsequently, 55 articles were assessed for eligibility through full-text review and 34 studies were finally included.

### 3.2 Quality Assessment of Included Studies

Quality assessment results of the included studies are shown in the Supplementary Materials (Table S2-4). 14 out of the 15 cross-sectional studies were of moderate quality (scores 4-7), 2 cohort studies were of high quality (a score of 8). Among the 22 animal studies, the risk of selective outcome reporting and other sources of bias was low. Most other items were not clearly reported, as follows: random sequence generation (21/22), baseline characteristics (12/22), allocation concealment (21/22), random housing (6/16), incomplete outcome data (18/22), blinding-detection bias (18/22), blinding-performance bias (18/22), and random outcome assessment (22/22).

### 3.3 Characteristics of Included Studies

As shown in Table 1, this study included 34 studies, comprising 4 combined clinical and animal studies, 12 clinical studies, and 18 animal studies. Among the clinical studies, there were 15 cross-sectional studies, 2 cohort studies, and 1 study with overlapping study design. Geographically, most publications originated from China (n = 20), followed by the United States (n = 6). The majority of the studies focused on biological cargos carried by CM-sEVs (n = 26) and the roles of CM-sEVs in intercellular communication (n = 24). A small number of studies investigated specific markers for CM-sEVs, *in vivo* tracing, and methodological advancements in detecting sEVs. In terms of disease types, a large proportion of studies focused on ischemic heart disease (11 clinical and 6 animal studies), followed by ischemia reperfusion (I/R) injury (1 clinical and 6 animal studies), and HF (3 clinical and 3 animal studies). Fewer studies investigated diabetic cardiomyopathy (1 clinical and 1 animal study), cardiac pressure overload (3 animal studies), atrial fibrillation (AF) (1 clinical and 1 animal study), doxorubicin (DOX)-induced cardiotoxicity (1 animal study), and aortic stenosis (AS) (1 clinical study). In the clinical studies, sample sizes ranged from 6 to 312 participants, including both male and female subjects, with age ranging from 35.48 to 86.34 years. Ultracentrifugation (UC) was the most widely used method for isolating sEVs, followed by precipitation-based kits or size exclusion chromatography (SEC). The characterization techniques for sEVs included nanoparticle tracking analysis (NTA) for particle size and concentration measurement, transmission electron microscopy (TEM) for morphological observation, and western blotting (WB) for the detection of positive and negative protein markers of sEVs to assess their purity and origin. Flow cytometry was used in several studies to precisely monitor the surface markers and analyze sEVs heterogeneity.

### 3.4 Validation Methods for Cardiomyocyte-Derived Small Extracellular Vesicles

Table 2 summarizes the validation strategies for the CM-sEVs in the 34 included studies. Among them, 31 studies combined *in vitro* and *in vivo* approaches. *In vitro* experiments were performed with primary cardiomyocytes, cardiomyocyte cell lines (H9c2, AC16, HL-1), and human induced pluripotent stem cell-derived cardiomyocytes (hiPSC-CMs). Based on cardiomyocyte cultures, 12 studies compared sEVs from different tissues or cell types to assist in confirming cardiomyocyte origin. These comparisons included secretion levels of sEVs, cargo molecules (e.g., miR-194-3p, miR-208a, and Cx43), and the expression of CM-sEVs origin-tracing markers (e.g., Ldb3, Ambra1, and CD172a). The control cells were cardiac fibroblasts (n = 8) and endothelial cells (n = 5). Twenty-four studies performed CM-sEVs tracing experiments. Among these, 5 studies combined *in vivo* and *in vitro* experiments, 14 studies conducted only *in vitro* experiments, and 5 studies conducted *in vivo* experiments. *In vivo* tracing approaches included cardiomyocyte-specific transgenic mice (αMHC/Myh6-MerCreMer-Rosa-mT/mG and TG-αMHC-STOP-CD63-NanoLuc), AAV-mediated labeling of cardiomyocyte-specific sEVs (AAV9-cTnT-CD63-GFP), metabolic labeling of cardiomyocyte-specific miRNAs (miR-208a), and fluorescent dye labeling (PKH67 and DiR). *In vitro* tracing was performed using fluorescent dyes and plasmid transfection of CD63-GFP fusion proteins. Some studies further identified CM-sEVs by evaluating cardiomyocyte-specific markers such as cTnT, α-MHC, and miR-208a or potential CM-sEVs markers such as Ldb3, CD172a, and Ambra1. Furthermore, several studies indirectly verified the cardiomyocyte origin of sEVs using genetic interventions (AAV9-mediated Ambra1 knockdown and β-arrestin 2 knockout) or exosome biogenesis inhibitors (GW4869 and DMA). One study used a peptide-anchored biomimetic interface combined with electrochemical impedance spectroscopy (EIS) and linear sweep voltammetry (LSV) for quantitative detection and specific validation of CM-sEVs *in vitro* and *in vivo*, thereby providing a novel approach for using electrochemical methods to determine the origin of sEVs.

### 3.5 Screening and Applications of Origin-Tracing Markers for the Cardiomyocyte-Derived Small Extracellular Vesicles

As summarized in Table 3, 15 studies reported cardiomyocyte-specific or CM-sEVs-specific markers, comprising 16 biomolecules. These markers were cTnT/Tnnt2/Troponin T (6 studies), CD172a, α-MHC/Myh6/MYH, and sarcomeric α-actinin (3 studies each), miR-208a and miR-1 (2 studies each), and Ambra1, Ldb3, cTnI, Cx43, α-SA, and miR-133a (1 study each). Most of these molecules are well-established cardiomyocyte-specific markers. Three studies systematically screened and validated Ldb3, CD172a, and Ambra1 as CM-sEVs origin-tracing markers, with CD172a and Ambra1 localized to the membrane of the sEVs. The screening and validation of markers spanned multiple sample types. At the cellular level, hiPSC-CMs, cardiomyocyte cell lines, and primary cardiomyocytes were evaluated under various conditions such as normoxia, hypoxia, high glucose/high lipid, hypoxia/reoxygenation (H/R), and other cellular stresses. Cardiac tissues as well as serum and plasma samples were collected and analyzed for the mouse models (normal, myocardial infarction, I/R, and transgenic) and a rapid-pacing canine model. Clinical studies analyzed serum and plasma samples from patients with acute myocardial infarction (AMI), ST-segment elevation myocardial infarction (STEMI), or HF, and ventricular tissue samples from ischemic HF patients.

Three studies comprehensively screened origin-tracing markers for the CM-sEVs. A study employed a two-stage proteomic strategy to screen for cardiac-derived EVs-associated markers. First, large extracellular vesicles (lEVs) and sEVs were isolated by differential UC from conditioned media of neonatal rat cardiomyocytes, cardiac fibroblasts, and the cardiomyoblast cell line H9c2. Comparative analysis revealed marked heterogeneity in EVs release levels and protein composition among different cardiac cell types, suggesting that distinct cardiac cell populations secrete EVs with characteristic molecular profiles, thereby providing a basis for subsequent source-marker identification. Based on these findings, EVs total recovery and purification (EVtrap) was further applied to enrich EVs from neonatal rat cardiac tissue and plasma, followed by liquid chromatography-mass spectrometry analysis. The results showed that Ldb3 was significantly enriched in cardiac-derived EVs compared with plasma EVs, suggesting that it may represent a candidate cardiac EVs-associated protein. Further analysis across multiple tissues (lung, liver, kidney, heart, plasma, skeletal muscle, brain, and skin) and cell types (cardiomyocytes and cardiac fibroblasts) indicated that Ldb3 was predominantly expressed in cardiac tissue and enriched in cardiomyocytes. In addition, multiple EVs isolation approaches, including UC, polyethylene glycol precipitation, and combined methods, consistently detected Ldb3 in both lEVs and sEVs, with relative enrichment in the sEVs fraction. SEC further confirmed that Ldb3 was mainly associated with EVs-enriched fractions rather than non-EVs components. Notably, although Ldb3 protein levels (78 kDa) were increased in the left ventricles of HF rats and patients with ischemic cardiomyopathy, no corresponding change was observed in circulating EVs fractions. This suggests that Ldb3 shows relatively stable detection levels in sEVs and supports its potential as a candidate origin-tracing marker for CM-sEVs 21.

Another study 38 found that sEVs secreted during I/R injury are associated with autophagy; moreover, secretion of the CM-sEVs is regulated by the interaction of a few genes with the autophagy-related protein LC3. Integrated analysis of gene datasets associated with cardiac fibrosis, vesicle formation, and autophagy, followed by GO/KEGG enrichment and network analyses, identified Ambra1 and Atg7 as potential candidates. Structural modeling and molecular docking further indicated that Ambra1, but not Atg7, possesses LC3-binding sites. Subsequently, Ambra1⁺ sEVs were isolated from H/R-treated neonatal mouse cardiomyocytes and from the myocardium of MI/R mice. Co-immunoprecipitation, WB, immunogold electron microscopy, and flow cytometry experiments confirmed that Ambra1 was localized on the vesicle membrane and co-localized with LC3. Functional experiments demonstrated that Ambra1 knockdown modulated both autophagy and the release of CM-sEVs. Ambra1⁺ sEVs were enriched with cardiomyocyte-specific markers such as cTnT and MHC. Moreover, Ambra1⁺ sEVs counts gradually decreased from the left ventricle to the aortic arch and peripheral circulation, further supporting their cardiac origin. Finally, WB analysis of different cardiac cell types (cardiomyocytes, fibroblasts, endothelial cells, and resident macrophages) and their corresponding sEVs demonstrated that Ambra1 expression was significantly high in the cardiomyocytes and cardiomyocyte-derived sEVs. Collectively, these findings suggested that Ambra1 was a promising membrane-specific marker of CM-sEVs.

CD172a is a surface marker of hiPSC-derived cardiomyocytes (hiPSC-CMs) 50. The expression of CD172a in hiPSC-CMs and adult myocardial tissues was first confirmed using confocal microscopy and immunohistochemistry. Subsequently, EVs were isolated from the hiPSC-CMs culture media by ultracentrifugation. Flow cytometry analysis demonstrated the presence of CD172a⁺ EVs (including sEVs). These CD172a⁺ EVs contained cardiomyocyte-specific proteins such as cTnT. The CM-derived EVs in human plasma were further analyzed. A reverse-gating strategy based on multicolor flow cytometry was used because of the coexistence of EVs from multiple tissue origins. Based on this, cellular debris and apoptotic bodies were first excluded, followed by EVs from erythrocytes, endothelial cells, platelets, leukocytes/lymphocytes, and monocytes. Finally, EVs expressing surface CD172a were positively selected as CM-EVs. Analysis of the internal cargos showed that more than 70% of plasma-derived CD172a⁺ EVs contained cTnT. Moreover, the number of CD172a⁺ EVs gradually decreased from the coronary sinus to the aortic arch and peripheral circulation, further supporting their myocardial origin 25.

In 15 studies, CM-sEVs-specific proteins were detected by WB (n = 5), flow cytometry (n = 4), RT-qPCR (n = 3), immunofluorescence (n = 2), and EIS combined with LSV (n = 1). Seven studies further investigated alterations in CM-sEVs-specific markers under CVDs conditions. Circulating levels of CD172a⁺ EVs were elevated in patients with AS, AMI, stable ischemic heart disease (SIHD), acute coronary syndrome (ACS), and hypertrophic cardiomyopathy (HCM), but the levels decreased a year after transcatheter aortic valve replacement (TAVR). Ambra1 expression was increased in the sEVs derived from hypoxia/reoxygenation-treated cardiomyocytes and in the serum sEVs from mice with I/R injury. Furthermore, miR-208a was significantly upregulated in both MI and chronic HF models, whereas the levels of α-MHC, miR-1, and Cx43 were elevated in the MI model mice. In contrast, Ldb3 (78 kDa) levels remained unchanged in the myocardial sEVs isolated from patients with ischemic HF.

Several studies confirmed the presence of cardiomyocyte-specific markers within sEVs but did not assess their alterations under disease conditions. For example, CD172a was identified in sEVs derived from hiPSC-CMs and H9c2 cells under physiological conditions, whereas Tnnt2 and Myh6 were detected in the cardiac tissues of C57BL/6N mice. Troponin T was also found in plasma sEVs isolated from cardiomyocyte-specific transgenic mice. Under pathological settings, Troponin T was detected in the plasma sEVs from patients with AMI and in myocardial tissues from the I/R model mice. Furthermore, α-sarcomeric actin (α-SA) was present in the sEVs released from primary cardiomyocytes exposed to high-glucose/high-lipid stress, and cTnI was identified in the atrial sEVs from rapid-pacing canine models.

### 3.6 Differential Changes in the Quantity and Cargos of CM-sEVs in Cardiovascular Diseases

#### 3.6.1 Differential Changes in the Quantity and Cargos of CM-sEVs in Ischemic Heart Disease

As summarized in Table 4, 11 clinical and 5 animal studies evaluated differential changes in the quantity and cargos of CM-sEVs in ischemic heart disease conditions. The clinical studies mainly focused on AMI and chronic myocardial ischemia, whereas most animal models were established by the permanent ligation of the left anterior descending (LAD) artery. Blood samples (plasma or serum) were the primary source of sEVs and only one study analyzed myocardial tissues. Most studies (10 out of 16) reported an increase in the circulating levels of sEVs and one study specifically demonstrated elevated levels of CD172a⁺ sEVs. Fourteen studies investigated the cargos in the CM-sEVs, focusing on alterations in the noncoding RNAs (9 studies) and proteins (5 studies). Overall, the levels of CD172a, HIF-1α, miR-30a, miR-208, miR-181a, and lncRNA HCG15 were upregulated and miR-24-3p was downregulated during myocardial infarction. Cx43 expression decreased during acute infarction but increased by day 15 post-MI. In chronic myocardial ischemia, CD172a, hERG1, and Hsp47 were significantly upregulated and miR-939-5p was downregulated. In patients with AMI, the expression of lncRNA HCG15 showed a strong positive correlation with cTnT and an Area Under the Curve (AUC) value of 0.952 in the Receiver operating characteristic (ROC) analysis indicated its high diagnostic value for AMI.

#### 3.6.2. Differential Changes in the Quantity and Cargos of CM-sEVs in Myocardial Ischemia/Reperfusion Injury

As summarized in Table 5, 1 clinical study and 6 animal studies evaluated differential changes in the quantity and cargos of CM-sEVs in myocardial I/R injury (MI/RI). The clinical study involved STEMI patients undergoing percutaneous coronary intervention (PCI), whereas the animal models of MI/RI were established by 30-60 min of LAD ligation followed by reperfusion. Most studies evaluated blood (plasma or serum) samples, whereas 2 studies additionally analyzed myocardial tissue samples. Among the 7 studies, 4 studies reported an increase in circulating levels of sEVs, including one study reporting elevated levels of Ambra1⁺ sEVs. All 7 studies evaluated the molecular cargos in the CM-sEVs. Clinically, miR-9-5p was upregulated in the serum sEVs-miR-9-5p of STEMI patients after PCI. In the animal models, the levels of Ambra1, mitochondrial DNA (mtDNA), and the miR-23-27-24 cluster were all increased after I/R, whereas the levels of miR-30d increased at 6-24 hours post-I/R but returned to baseline at 48 h. The levels of Cx43 decreased 30 minutes after I/R but recovered within 4 hours. Moreover, treatment with the traditional Chinese medicine Tongxinluo (TXL) alleviated I/R injury by upregulating linc-ROR in the CM-sEVs. Furthermore, CM-sEVs derived from healthy mice carried ATP5a1, which attenuated oxidative stress and cardiac dysfunction induced by MI/RI.

#### 3.6.3 Differential Changes in the Quantity and Cargos of CM-sEVs in Valvular Heart Disease, Cardiomyopathy, and Cardiac Arrhythmias

As shown in Table 6, 3 clinical studies and 6 animal studies evaluated differential changes in the quantity and cargos of CM-sEVs in valvular heart disease, cardiomyopathy, and cardiac arrhythmias. The clinical studies involved patients with diabetic cardiomyopathy, AF, AS, and hypertrophic cardiomyopathy. The animal models comprised a rapid atrial pacing canine model, DOX-induced cardiotoxicity model, cardiomyocyte-specific gene-edited mice mimicking cardiac remodeling and hypertrophy, transverse aortic constriction (TAC) model mice and pigs to induce pressure overload, and a streptozotocin (STZ)-induced diabetic cardiomyopathy model mice. Among the included studies, 6 studies evaluated blood samples, whereas 3 studies analyzed cardiac tissue samples. Eight studies reported increased sEVs levels in both clinical and animal models. The levels of sEVs were elevated during early compensatory hypertrophy (5 days post-TAC) and declined during late decompensation (9 weeks post-TAC). The CD172a⁺ EVs levels were significantly elevated in AS patients but declined one year after TAVR. This reduction was already detectable two months post-TAVR in a cohort of severe AS patients, suggesting an early postoperative response. Seven studies further characterized the molecular cargos of CM-sEVs. In diabetic cardiomyopathy, serum sEVs from patients exhibited downregulation of miR-194-3p, whereas in murine models of diabetic cardiomyopathy, the expression of Hsp20, SOD1, survivin, and p-Akt was upregulated. Furthermore, plasma CD172a⁺ EVs from AS patients showed elevated ceramide levels, and serum miR-210-3p expression was also increased in AF. In a study using the TAC model, myocardial expression of Cryab exhibited a dynamic pattern, with significant upregulation during the early compensatory phase (5 days post-TAC) but a marked decline in the late decompensated stage (9 weeks post-TAC). Furthermore, single-cell transcriptomic analysis in the inducible cardiomyocyte-specific β-catenin gain-of-function mice (mimicking cardiac remodeling and hypertrophy) demonstrated elevated expression of Cryab and increased levels of several proteostasis-related proteins (HSPA70, HSP90AB1, VCP, UBE2N, PKACA, and BAG2).

This was accompanied by enhanced ubiquitination, upregulation of 20S proteasome α/β subunits, and increased expression of Z-disc structural proteins (DES, DMD, MYBPC3, MYL2, TTN, and PLN). However, these protein changes have not yet been experimentally validated. ROC analysis demonstrated that circulating cardiac CD172a⁺ EVs yielded an AUC of 0.768 with a sensitivity of 67.3% and specificity of 65.3%, thereby indicating a moderate diagnostic performance for AS. Moreover, pre-TAVR CD172a⁺ EVs levels positively correlated with 1-year survival, thereby suggesting a positive prognostic role.

#### 3.6.4 Differential Changes in the Quantity and Cargos of CM-sEVs in Heart Failure

As summarized in Table 7, 6 studies—3 clinical and 3 animal investigations—evaluated differential changes in the quantity and cargos of CM-sEVs in HF. The clinical studies focused on HF patients, whereas animal models included a mouse model of HF induced by Ang II, a mouse model of chronic ischemic HF established 4 weeks after I/R, and a rat model of chronic HF established 6 weeks after MI. Furthermore, 3 studies analyzed blood-derived samples and 2 studies evaluated left ventricular tissues. Two studies evaluated quantitative changes in the sEVs. In one clinical study, sEV levels were elevated in patients with compensated HF but reduced in those with decompensated HF. Another study reported higher sEVs levels in the Ang II-treated mice. All 6 studies investigated the molecular cargos in the CM-sEVs. A clinical study reported that hERG1 and Hsp47 were upregulated in patients with compensated HF but downregulated in those with decompensated HF. Moreover, the Hsp47/hERG1 ratio was significantly decreased. This suggested that alterations in the cargos composition of the sEVs may serve as a potential biomarker for distinguishing HF subtypes. Another clinical study reported that plasma miR-30d-5p levels were reduced in patients with ischemic HF. This was consistent with findings in a chronic ischemic HF mouse model. In animal studies, miR-21-5p and miR-208a were both upregulated in a rat model of chronic HF at 6 weeks post-MI, whereas Ang II-induced HF was associated with activation of the Sonic hedgehog (Shh) signaling pathway. Ldb3 was markedly decreased in the myocardial tissue of patients with ischemic cardiomyopathy-related HF, whereas it remained relatively stable in sEVs derived from the same myocardium.

### 3.7 Intercellular Communication Mediated by the CM-sEVs

#### 3.7.1 CM-sEVs-Mediated Communication Between Cardiomyocytes

As summarized in Table 8, 7 studies investigated CM-sEVs-mediated intercellular communication among cardiomyocytes. The CM-sEVs were derived from cardiomyocyte cell lines or primary cardiomyocytes. Three studies used hypoxic stimulation, whereas others used high glucose, Ang II, or DOX treatment. Furthermore, 1 study examined CM-sEVs from healthy mice and demonstrated that their cargos protected cardiomyocytes subjected to I/R injury. All studies performed *in vitro* tracing of CM-sEVs using CD63-GFP fusion proteins or membrane dyes such as PKH67 and DiO, whereas a few studies combined both *in vivo* and *in vitro* labeling approaches. The pathological processes were mainly related to oxidative stress, autophagy, apoptosis, and inflammation. In hypoxic conditions, CM-sEVs showed elevated levels of miR-30a, which suppressed autophagy and promoted apoptosis and inflammatory responses by targeting Beclin-1, ATG5, and ATG12 in the recipient cardiomyocytes. Furthermore, hypoxia-induced upregulation of lncRNA HCG15 in the CM-sEVs activated NF-κB/p65 and p38 MAPK signaling, thereby aggravating ischemic injury by promoting cardiomyocyte apoptosis and inflammation. In contrast, CM-sEVs derived from healthy mice delivered ATP5a1 to the cardiomyocytes exposed to H/R and reduced mitochondrial ROS production, alleviated mitochondrial damage, and inhibited ferroptosis. Furthermore, CM-sEVs released from DOX-treated cardiomyocytes exacerbated oxidative stress by increasing ROS generation and suppressing antioxidant defenses. Ang II stimulation contributed to cardiomyocyte hypertrophy by promoting the release of CM-sEVs enriched in Shh/N-Shh/Gli1. In diabetic conditions, CM-sEVs exhibited significantly reduced Hsp20 levels, whereas transgenic Hsp20 overexpression in primary cardiomyocytes increased Hsp20, SOD1, survivin, and p-Akt in the sEVs, which mitigated high glucose-induced oxidative stress and preserved cardiomyocyte function.

#### 3.7.2 CM-sEVs-Mediated Communication Between Cardiomyocytes and Fibroblasts

As summarized in Table 9, 6 studies reported on CM-sEVs-mediated communication between cardiomyocytes and fibroblasts. The CM-sEVs were derived from primary cardiomyocytes. All studies conducted sEVs-tracing experiments. The fluorescent dye PKH67 was commonly used for labeling. A study used molecular beacons for real-time imaging of intercellular and sEVs-encapsulated miRNAs, and subsequently employed a Cre-reporter mouse model to visualize transfer and uptake of CM-sEVs *in vivo*. The recipient cells were tracked via GFP signals to provide direct evidence for CM-sEVs-mediated intercellular communication. In pathological conditions, CM-sEVs contributed to cardiac fibrosis by modulating fibroblast functions. Specifically, miR-24-3p was downregulated in CM-sEVs under ischemia-simulating conditions, leading to upregulation of its target genes such as FURIN, CCND1, and SMAD4, which activate TGF-β signaling and promote fibroblast proliferation and differentiation into myofibroblasts. During high glucose/high lipid conditions, miR-194-3p levels were reduced in the CM-sEVs, leading to enhanced TGFβR2 expression, activation of the TGFβ-Smad pathway, fibroblast activation, and fibrosis. In contrast, under hypoxic conditions, CM-sEVs were enriched in miR-30d-5p, which target Itga5 to attenuate TGF-β/Smad signaling and suppress fibrotic responses. Ang II stimulated the release of CM-sEVs enriched in Shh/N-Shh/Gli1, which drive fibroblast activation and proliferation by activating the Shh signaling pathway. In a H/R injury model, Ambra1^+^ sEVs transfer mtDNA from injured cardiomyocytes to fibroblasts, thereby triggering cGAS-STING signaling and promoting fibroblast proliferation. Furthermore, atrial CM-sEVs enriched in miR-210-3p enhanced fibroblast proliferation and collagen production via the GPD1L/PI3K/AKT pathway.

#### 3.7.3 CM-sEVs-Mediated Communication Between Cardiomyocytes and Endothelial Cells

As shown in Table 10, 5 studies reported that CM-sEVs mediated the communication between cardiomyocytes and endothelial cells. Most CM-sEVs were derived from primary cardiomyocytes. Three studies performed tracing experiments with PKH26 or PKH67 fluorescent dyes *in vitro*. Functionally, CM-sEVs regulated key pathological processes—including angiogenesis, cell proliferation, oxidative stress, and apoptosis—by transferring specific cargos to target endothelial cells. For example, sEVs with low miR-143 levels promoted NO production and enhanced angiogenesis in HUVECs by relieving the suppression of its target gene IGF-IR. H/R-treated cardiomyocytes preconditioned with TXL secrete sEVs enriched with linc-ROR, which inhibits miR-145-5p in the cardiac microvascular endothelial cells, enhances eNOS phosphorylation and NO production, and mitigates oxidative stress and apoptosis. Similarly, Hypoxia-treated neonatal rat CM-sEVs with low miR-939-5p levels promoted endothelial cell proliferation and angiogenesis by activating the iNOS-NO signaling pathway. In addition, HL-1 cardiomyocyte-derived sEVs, when co-cultured with primary endothelial cells, increased MMP3 secretion and promote cell migration and proliferation; however, excessive MMP3 activity leads to extracellular matrix degradation, impaired tube formation, and endothelial cell death. Furthermore, sEVs from HSP20-transgenic cardiomyocytes alleviated high glucose-induced oxidative stress in the endothelial cells.

#### 3.7.4 CM-sEVs-Mediated Communication Between Cardiomyocytes and Other Cell Types

As shown in Table 11, 9 studies investigated the mechanisms by which CM-sEVs mediate communication between cardiomyocytes and various cell types. On the heart-brain axis, CM-sEVs were shown to interact with neurons (1 study) and microglia (1 study). On the heart-immune axis, CM-sEVs regulate macrophages (4 studies) and neutrophils (1 study). On the heart-adipose axis, CM-sEVs affect adipocytes (1 study). On the heart-lung axis, CM-sEVs influence pulmonary endothelial cells (1 study). Among these studies, most performed *in vitro* tracing experiments with CM-sEVs using the fluorescent dye PKH26. In addition, more advanced approaches have been employed, including genetic reporter systems (Myh6-Cre/Rosa-mT/mG mice), viral labeling for *in vivo* tracking (AAV9-cTnT-CD63-GFP), and cell type-specific metabolic labeling combined with sEVs miRNA tracking, allowing precise identification of CM-sEVs origin and cargos delivery *in vivo*. CM-sEVs mediated inter-organ communication under pathological conditions, affecting processes such as inflammation, ER stress, and apoptosis.

On the heart-brain axis, CM-sEVs enriched in miR-21-5p promoted M1 microglial polarization and neuroinflammation in MI-induced HF by suppressing PRMT1, contributing to cardiogenic dementia, while in MA-induced conditioned place preference in rats, CM-sEVs carrying miR-181a-5p crossed the blood-brain barrier, were internalized by SH-SY5Y cells, and increased miR-181a-5p levels in the brain. On the heart-lung axis, CM-sEVs from MI mice were enriched in miR-208a, which targeted NLK and Tmbim6 in lung endothelial cells, activated NF-κB signaling, and induced pulmonary inflammation and structural remodeling. On the heart-immune axis, CM-sEVs from neonatal mice subjected to H/R exhibited upregulated miR-9-5p, which regulated N1 neutrophil polarization via the SIRT1/NF-κB and SOCS5/JAK2/STAT3 pathways, and HL-1 cardiomyocytes under hypoxia or rapid pacing secreted sEVs that induced M1 polarization of RAW264.7 macrophages, collectively amplifying inflammatory responses. Finally, on the heart-adipose axis, MI/R-induced CM-sEVs enriched in the miR-23-27-24 cluster were taken up by adipocytes, suppressed EDEM3, activated ER stress via PERK/ATF6 pathways, reduced adiponectin production, triggered endocrine dysregulation, and established a deleterious feedback loop between the heart and adipose tissue.

## 4. Discussion

CM-sEVs carry a variety of biomolecules reflecting the pathophysiological state of their source cells or tissues and mediate intercellular communication by transferring signals. Therefore, CM-sEVs are promising biomarkers for CVDs. However, since the origins of circulating sEVs are heterogeneous, accurate tracing of the CM-sEVs is important. To date, most studies on CM-sEVs have been conducted *in vitro* using cultured cardiomyocytes and lack *in vivo* validation and comparative analyses with sEVs from other cell types. Hence, they may not fully capture the physiological and pathological alterations of cardiomyocytes *in vivo*. To address these limitations, we performed a systematic review of *in vivo* studies on CM-sEVs in both patient and animal models.

In this systematic review, we first summarized the methods used for identifying the CM-sEVs and their characteristic molecular markers across the 34 included studies. The identification of CM-sEVs was mainly based on *in vitro* cultured cardiomyocytes combined *in vitro* and *in vivo* tracing of CM-sEVs, as well as identifying specific molecular markers of CM-sEVs. In most studies, cardiomyocyte-specific proteins such as cTnT and Myh6 were used to determine the cardiomyocyte origin of sEVs. Moreover, 3 studies systematically investigated CM-sEVs-origin-tracing markers, including Ldb3, Ambra1, and CD172a to reflect the unique association between CM-sEVs and cardiomyocytes and enable their accurate identification. Second, we summarized the changes in the abundance and cargos of CM-sEVs under various CVD conditions, including ischemic heart disease (myocardial infarction and myocardial ischemia), I/R injury, structural heart disease (arrhythmias, valvular heart disease, cardiomyopathies), and HF. This highlighted their potential as clinical biomarkers for early diagnosis and prevention of CVDs. Finally, we collated evidence that CM-sEVs mediate communication not only between cardiomyocytes and other cardiac cell types but also across distant organs, highlighting their role in orchestrating intercellular signaling and influencing systemic responses under pathological conditions.

### 4.1 Potential Origin-tracing Markers of CM-sEVs

Since myocardial tissue cannot be directly obtained from patients, identifying bioactive molecules in the blood that reflect pathological changes in cardiomyocytes is highly significant for the early prediction, diagnosis, and prognosis of CVDs and their associated complications. However, peripheral blood contains mixed signals from multiple tissues, complicating the specific identification of cardiomyocyte-derived changes 51,52. The sEVs reflect the molecular characteristics of their cells of origin. For example, neuron-derived sEVs in blood carry Aβ, Tau, synaptic proteins, and specific miRNAs, and possess high diagnostic and predictive value for Alzheimer's disease 53. Therefore, it is critical to identify CM-sEVs in circulation. Markers such as caveolin-3 and CPT1B have been proposed for cardiac-derived EVs, but their cell-type specificity within the heart remains unclear 54,55. Similarly, EVs isolated from Langendorff-perfused hearts contain proteins from cardiomyocytes, fibroblasts, endothelial cells, immune cells, and smooth muscle cells 56, reflecting high cellular heterogeneity. In addition, sample type (plasma vs. serum) and isolation methods can further influence EVs composition and bias CM-sEVs identification 51. Collectively, these challenges make it difficult to accurately distinguish CM-sEVs in peripheral blood. Currently, there are no universally recognized specific markers for identifying the CM-sEVs. Among the included studies in this investigation, 3 studies systematically screened and identified Ldb3, Ambra1, and CD172a as potential origin-tracing markers of CM-sEVs 21,25,38.

Ldb3 (also known as Cypher or ZASP) is a Z-disc-associated protein in striated muscle that plays an important role in sarcomere stability and cytoskeletal remodeling 57. The studies included in this review showed that, compared with multiple tissues from neonatal rats (including lung, liver, kidney, plasma, skeletal muscle, brain, and skin), Ldb3 is predominantly expressed in cardiac tissue; it is also enriched in cardiomyocytes compared with neonatal cardiac fibroblasts. Notably, although Ldb3 exhibits multiple alternatively spliced isoforms in cardiac tissue, only a single protein form of approximately 78 kDa is predominantly detected in the analyzed sEVs, and its overall level remains relatively stable in sEVs derived from patients with ischemic cardiomyopathy 21. These findings suggest that Ldb3 may reflect compositional features of CM-sEVs and has potential as a candidate origin-tracing marker for CM-sEVs. It should be noted that existing evidence also indicates that Ldb3 is expressed in skeletal muscle and the nervous system 57,58, and the distribution of different Ldb3 splice isoforms across tissue-derived sEVs has not yet been systematically characterized. Therefore, the specificity of Ldb3 as a CM-sEVs origin-tracing marker still requires further validation in multi-tissue sEV systems, particularly at the level of isoform resolution.

Ambra1, a classical regulator of autophagy 59, is significantly enriched on the surface of CM-sEVs membranes under MI/R injury conditions. Among sEVs derived from distinct types of cardiac cells, Ambra1 expression was most prominent in those from cardiomyocytes. Functional experiments further demonstrated that Ambra1 affects both vesicle biogenesis and uptake, thereby suggesting that it may be involved in mediating vesicle-cell interactions. Moreover, Ambra1 as a vesicular component protein is associated with myocardial fibrosis and mitophagy following MI/R injury. Ambra1 primarily exists in complex with the autophagy-related protein LC3. Therefore, combined detection of Ambra1 and LC3 could be used to identify the *in vivo* generation of CM-sEVs generated during I/R injury 38. Since Ambra1 is localized on the vesicle membrane surface, it may also participate in the selective recognition of recipient cells or organ-specific homing. However, this mechanism remains to be elucidated. Furthermore, dysregulation or mutation of Ambra1 is implicated in various systemic diseases, including neurological disorders 60, cancers 61, anemia 62 and enteritis 63. Because of the lack of comparative data on sEVs across different organs, it is not clear whether Ambra1 is a specific protein in the CM-sEVs.

The transmembrane protein CD172a (also known as SIRPα) was initially recognized as a marker of immune cells 64-66. However, recent studies have demonstrated its expression in hiPSC-CMs and human fetal cardiomyocytes. Using an anti-CD172a antibody to sort hiPSC-CMs enables the isolation of a cell population with up to 98% cTnT-positive cardiomyocytes 50. In the studies included in this review 25, CD172a⁺ EVs were enriched in cardiomyocyte-specific molecules such as cTnT and miR-1, and their abundance gradually decreased from the coronary sinus to the peripheral circulation, thereby supporting their cardiac origin. Multicolor flow cytometry of peripheral blood samples combined with a reverse exclusion strategy demonstrated good specificity and effective discrimination of CM-EVs from those originating from other cell types. Furthermore, another study used a peptide-anchored electrochemical sensing method targeting CD172a, and achieved efficient enrichment and detection of CM-EVs in serum samples from patients with AMI, thereby exhibiting significant clinical diagnostic potential 20. It is noteworthy that EVs represent a heterogeneous population comprising vesicles of different sizes. Therefore, CD172a⁺ EVs detected in the above studies may include not only sEVs but also EV subpopulations of other sizes. Accordingly, these findings more likely reflect the overall characteristics of the CD172a⁺ EV population, rather than being strictly equivalent to CM-sEVs. The specific subpopulation composition and origin specificity of CD172a⁺ EVs still require further clarification using higher-resolution isolation strategies and additional experimental validation.

In addition to the 3 studies mentioned above, several other investigations have used cardiomyocyte markers such as miR-208a, cTnT/Tnnt2, and α-MHC/Myh6 to identify and confirm sEVs originating from the cardiomyocytes. The miR-208 family is a group of key miRNAs specifically expressed in the cardiac tissues. MiR-208a and miR-208b are located within the introns of the α- and β-myosin heavy chain genes (Myh6 and Myh7), respectively, and miR-208a is highly specific to cardiomyocytes 67,68. In a mouse model, cardiomyocyte-specific UPRT labeling was used to tag newly synthesized miR-208a and miR-1 with significantly higher efficiency in cardiac tissue compared to other organs, and both miRNAs were detected in peripheral blood sEVs. MiR-1 is also expressed in skeletal muscle, but miR-208a expression is exclusively restricted to the heart. Therefore, detection of miR-208a in blood or sEVs serves as a reliable indicator of cardiomyocyte origin 42. Troponin T, a component of the troponin complex, regulates actin-myosin interactions and muscle contraction, and is expressed in both cardiac and skeletal muscles 69. The cardiac isoform cTnT is expressed in the cardiomyocytes and is widely used clinically to detect myocardial injury 70. Tnnt2, the gene encoding cTnT, is highly specific to the cardiac muscle 71. Therefore, cTnT and Tnnt2 are used as molecular marker to validate the origin of the CM-sEVs and reflect myocardial pathological states. Myh6 encodes α-myosin heavy chain (α-MHC), a core contractile protein responsible for myocardial contraction 72. In adult mammals, Myh6 is abundantly expressed in the atrial and ventricular myocardium and is a well-established cardiomyocyte marker associated with ischemic heart disease 73. Therefore, it is a potential indicator for identifying CM-sEVs. However, Tnnt2 has also been reported to be highly expressed in the plasma of patients with amyotrophic lateral sclerosis, which may limit its specificity 74. Moreover, several other biomolecules related to myocardial structure or function, such as miR-1 and miR-133a, are also present in skeletal muscle and limit their ability to definitively distinguish CM-sEVs 75,76. Therefore, using a single marker may not be sufficient to accurately define the cardiomyocyte origin of sEVs. A multi-marker detection approach or integration of tracing techniques and multi-omics analyses would provide greater specificity and reliability.

### 4.2 The Potential Value of CM-sEVs-Derived Molecules as Biomarkers for CVDs

Twenty-four studies reported changes in the quantity or cargos of CM-sEVs in CVDs. Overall, the quantity of CM-sEVs increases under disease conditions. A few studies suggested that the secretion of CM-sEVs exhibited distinct stage-dependent characteristics. Both nucleic acids and proteins carried by the CM-sEVs show stage-specific variations, thereby highlighting their potential value in disease diagnosis and prognosis assessment. Noncoding RNAs such as lncRNAs and miRNAs have emerged as key molecules in cardiovascular research in recent years. LncRNAs regulate gene expression through multiple mechanisms at the transcriptional and post-transcriptional levels and participate in critical biological processes of the cardiomyocytes such as growth, differentiation, apoptosis, and fibrosis 77-79. miRNAs mediate post-transcriptional silencing by binding to the target mRNAs and fine-tuning the stress responses, energy metabolism, and intercellular communication of the cardiomyocytes 80-82. LncRNA HCG15 levels were significantly higher in sEVs isolated from AMI patients and hypoxic cardiomyocytes than in controls, and its expression correlated strongly with cTnT concentration, thereby suggesting its potential as an early diagnostic biomarker for MI (AUC = 0.952) 26. In the early phase of MI/R injury, CM-sEVs carrying miR-9-5p induce N1 polarization of neutrophils and aggravate cardiac dysfunction. Furthermore, miR-9-5p levels are independently associated with cardiovascular mortality in STEMI patients undergoing PCI and demonstrate good clinical prognostic value (AUC = 0.67) 17. The miR-30 family is one of the most abundant miRNAs in the heart 83. Multiple studies have reported dynamic changes of miR-30 family members during different stages of MI 24,31,34,43. Hypoxia induces upregulation of miR-30a in cardiomyocytes and its enrichment in the CM-sEVs 31. Furthermore, miR-30a levels are significantly elevated in the serum sEVs of AMI patients and AMI model mice potentially due to autophagy inhibition 31,34. The expression levels of miR-30d increase during the acute phase after ischemic remodeling and exert protective effects, but decrease during the chronic-phase and are associated with adverse cardiac remodeling. In rodent models and chronic HF patients, higher miR-30d levels are linked to beneficial cardiac remodeling 24. This suggests that members of the miR-30 family may serve as potential biomarkers for monitoring acute cardiac injury and chronic remodeling. MiR-208a, an auxiliary marker for identifying CM-sEVs, was significantly increased in the peripheral blood sEVs from the MI model mice with pulmonary complications and in the left ventricular sEVs from rats with chronic HF 32,42. MiR-208a levels in the lung tissues peak at 24 h after MI, and act by suppressing the expression of inflammation-regulating factors NLK and Tmbim6 in the pulmonary cells, thereby contributing to MI-associated pulmonary complications 42.

At the protein level, the quantity of CM-sEVs demonstrates dynamic changes during myocardial injury and repair. CD172a, a potential specific biomarker of CM-sEVs, is partially released from the heart during myocardial hypoxia and enhances positive inotropic effects in the non-ischemic cardiac regions. CD172a⁺ EVs are significantly elevated in patients with AS and are also increased in the circulation of patients with ischemic heart disease and cardiomyopathy. Hypoxia is a key promoting factor for the release of CD172a⁺ EVs from cardiomyocytes. The preoperative circulating CD172a⁺ EVs levels in AS patients positively correlate with postoperative outcomes 25. These findings suggest that CD172a may not only serve as a potential CM-sEVs marker, but also the abundance of CD172a⁺ vesicles may also have potential diagnostic (AUC = 0.768) and prognostic value in AS. Ambra1, a vesicular protein associated with myocardial fibrosis and mitophagy following MI/R, is significantly increased by approximately fivefold in the CM-sEVs from MI/R hearts. Ambra1⁺ sEVs carrying mitochondrial components are internalized by fibroblasts and participate in the activation of fibrotic factors and the cGAS-STING pathway, thereby promoting fibroblast activation and proliferation 38. These findings highlight the need for further experimental and clinical studies to clarify the relationship between Ambra1 levels on the membranes of CM-sEVs and CVDs. Ldb3, another potential specific marker of CM-sEVs, is significantly decreased in the myocardial tissues of patients with ischemic HF but remains stable in the sEVs derived from patient myocardial tissue 21. This suggests that Ldb3 is associated with the formation and secretion of CM-sEVs and may serve as a structural or origin-specific marker of CM-sEVs. Cx43, a key structural component of cardiomyocyte gap junctions 84, not only facilitates synchronized electrical impulse conduction throughout the heart and directly mediates intercellular communication within cardiac tissue 85, but also participates in regulating communication between sEVs and recipient cells. Studies have shown that circulating Cx43 levels are significantly decreased in patients during AMI and in animal models at the acute phase of I/R (30 min), whereas they are elevated in murine models at 15 days after MI. This time-dependent pattern suggests that Cx43 may reflect the dynamic remodeling process during myocardial injury and repair. Under basal conditions, ubiquitination of Cx43 promotes its sorting into multivesicular bodies and subsequent release via sEVs. Under ischemic stress, however, this sorting process is disrupted, and Cx43 is preferentially targeted for degradation through the p62-mediated autophagic pathway, leading to reduced Cx43 levels in sEVs. At later stages, the rebound of Cx43 levels may be associated with myocardial structural remodeling and restoration of intercellular communication during gap junction regeneration 27,47.

In summary, CM-sEVs in blood samples not only reflect myocardial injury during the acute phase but also participate in ventricular remodeling. Therefore, they provide new molecular evidence for the early diagnosis and prognostic assessment of CVDs.

### 4.3 Intercellular Communication Mediated by CM-sEVs

Cardiomyocytes are not only the mechanical units of the heart but also key signaling hubs that regulate cardiac homeostasis. In recent years, several studies have demonstrated that cardiomyocytes secrete sEVs with bioactive components to communicate with nearby or distant cells, thereby exerting multi-level regulatory effects during cardiac injury, repair, and remodeling 86, 87. The evidence summarized in this study indicates that CM-sEVs participate in both local intercellular communication within the myocardium and long-range communication between the heart and distal organs (e.g., heart-lung, heart-brain, and heart-adipose tissue axes). The tracing methods for CM-sEVs primarily involve fluorescent dyes, fusion proteins, and genetic labeling techniques. The biological effects of CM-sEVs involve regulation of key processes such as myocardial fibrosis, inflammatory responses, angiogenesis, oxidative stress, and cell death.

In the research regarding CM-sEVs, tracing techniques are necessary to elucidate their origin, targeting, and roles in intercellular communication. Lipophilic dyes (e.g., PKH26, PKH67, DiR) are the most widely used tracing agents for the *in vitro* detection of sEVs released by primary cardiomyocytes or cell lines and taken up by fibroblasts, endothelial cells, or immune cells; *in vivo* applications enable non-invasive imaging of labeled exosomes 19,22,37. This method is simple to perform but is prone to interference from non-specific adsorption 88. In contrast, sEVs markers (such as CD63) tagged with fluorescent proteins allow more stable and specific tracing, and targeted expression in the cardiomyocytes via viral vectors more accurately reflects the origin and targeting of sEVs 31,34,41,46. Cre-loxP or genetic labeling systems enable single-cell-level tracking of transfer and functional changes in the CM-sEVs, thereby facilitating the analysis of their signaling roles in intercellular communication 24,32,42. Overall, fluorescent dyes are suitable for observing macroscopic distribution, fusion protein labeling improves specificity, and genetic labeling supports mechanistic studies. Combining these approaches provides a multidimensional analysis of CM-sEVs dynamics within the cardiac microenvironment and between distal organs.

CM-sEVs-mediated intercellular communication exhibits a distinct bidirectional nature. Pathological stimuli such as hypoxia, DOX, or Ang II induce cardiomyocytes to release sEVs enriched with various bioactive molecules that promote inflammation, inhibit autophagy, and induce cell death, thereby exacerbating myocardial injury 26,31,34. Conversely, physiological or protective stimuli can trigger secretion of sEVs carrying antioxidant and mitochondrial protective signals, thereby mitigating stress responses 37,48. This “detrimental-protective” dual effect suggests that sEVs are not merely byproducts of cellular damage, but rather active signaling carriers through which cardiomyocytes regulate neighboring and distant cell responses under different conditions. In cardiomyocyte-cardiomyocyte communication, CM-sEVs modulate autophagy, oxidative stress, and inflammatory states, and participate in both self-protection and the propagation of injury within the myocardium 34,35,37. In cardiomyocyte-fibroblast communication, myocardial fibrosis represents the major pathological event. Most studies have focused on the CM-sEVs mediating activation, migration, and collagen synthesis of fibroblasts via the TGF-β signaling pathway 16,19,24, but the regulatory direction is not always consistent. This suggests that the influence of cardiomyocytes on fibroblasts is dynamic. Such discrepancies may stem from differences in stimulus intensity, disease stage, or metabolic status. In cardiomyocyte-endothelial cell communication, the main pathological processes involve angiogenesis. CM-sEVs enhance NO signaling and promote neovascularization through molecules such as miR-143 and miR-939-5p 8,30. In immune regulation, sEVs mediate bidirectional polarization of macrophages, thereby balancing inflammatory clearance and tissue repair 29,32. These phenomena indicate that CM-sEVs do not act in isolation on a single target cell type but rather orchestrate the reconstruction of cardiac homeostasis by modulating endothelial integrity, immune activation, and extracellular matrix remodeling. Moreover, the effects of CM-sEVs extend beyond the heart, reflecting a systemic “heart-organ communication” network. For example, miR-208a can cross the heart-lung axis via sEVs to induce pulmonary endothelial inflammation 42, whereas miR-21-5p can enter microglial cells after HF and trigger neuroinflammation and cognitive impairment 32. Furthermore, MI/R-induced sEVs can disrupt lipid metabolism and endocrine function in adipose tissues 41. Collectively, these findings suggest that CM-sEVs may serve as key mediators through which the heart transmits pathological signals to distant organs, thereby providing new mechanistic insights into multi-organ comorbid injury.

## 5. Strengths and Limitations

This systematic review focusing on CM-sEVs revealed that Ldb3, CD172a, and Ambra1 are potential specific molecular markers of CM-sEVs. These markers can be used to trace the origin of CM-sEVs and serve as potential diagnostic biomarkers for diseases based on peripheral blood, with broad clinical application prospects. These studies also offer a research paradigm for identifying markers of sEVs from different cellular sources. Furthermore, cardiomyocyte-specific markers such as miR-208a, cTnT/Tnnt2, and α-MHC/Myh6 can be used to aid in the identification of CM-sEVs. The proteins and ncRNAs carried by CM-sEVs show dynamic changes across different CVDs, highlighting their potential clinical value in early diagnosis, disease monitoring, and prognosis evaluation. Moreover, CM-sEVs mediate communication among cardiomyocytes, fibroblasts, endothelial cells, and immune cells, thereby highlighting the molecular crosstalk underlying cardiac injury and repair. However, these potential characteristic markers of CM-sEVs may partially overlap with those of sEVs derived from skeletal muscle or immune cells. Therefore, further validation is required. In addition, effective *in vivo* isolation methods for CM-sEVs are lacking, and the dynamic changes and regulatory mechanisms of CM-sEVs cargos during different disease stages remain unclear. These limitations restrict the precise diagnostic and therapeutic application of CM-sEVs in CVDs.

## 6. Conclusion

This systematic review of 34 studies summarized the specific markers of CM-sEVs, the alterations in their molecular cargos, and their roles in mediating intercellular communication. Sixteen molecules, including Ldb3, CD172a, and Ambra1 were identified as potential origin-tracing markers of CM-sEVs. These studies also provide a methodological paradigm for identifying specific biomarkers for cell type-specific sEVs. Well-established cardiomyocyte markers such as miR-208a, cTnT/Tnnt2, and α-MHC/Myh6 can serve as auxiliary markers for identifying CM-sEVs. Some of these characteristic markers are associated with CVDs. For example, CD172a⁺ EVs are significantly elevated in the circulation of patients with aortic valve stenosis; Ambra1⁺ sEVs are increased in mice with MI/R injury; and Ldb3 is markedly reduced in myocardial tissue from HF patients but remains stable in myocardial tissue-derived sEVs. MiR-208a is significantly upregulated in circulating sEVs from animal models of MI complicated by pulmonary injury and chronic HF. Three studies reported potential diagnostic applications for the CM-sEVs. Higher CD172a⁺ EVs counts before TAVR predicted better prognosis for patients with AS; serum sEVs-related miR-9-5p levels were negatively correlated with LVEF and independently associated with cardiovascular mortality in STEMI patients within 24 h after PCI; and serum sEVs-related lncRNA HCG15 was significantly upregulated in AMI patients, thereby suggesting potential diagnostic value for early MI. Moreover, miR-30a, miR-30d, and Cx43 levels in circulating sEVs from patients and animal models exhibit dynamic alterations across different CVDs. In terms of intercellular communication, CM-sEVs mediate signaling not only between cardiomyocytes and other cardiac cell types (e.g., fibroblasts, endothelial cells) but also with distant organs such as the brain, lungs, and adipose tissue. Consequently, CM-sEVs contribute to the regulation of cardiac fibrosis, autophagy, oxidative stress, angiogenesis, immune-inflammatory responses, and inter-organ pathological interactions. These findings provide important insights into CM-sEVs origin identification, disease biomarker discovery, and intercellular communication mechanisms, supporting their potential translational application in cardiovascular diseases.

## Supplementary Material

Supplementary tables.

## Figures and Tables

**Table 1:** Characteristics of Included Studies

Author, year, country	Study designs	Diseases/Models	Groups (sample size)	Male (%)/Sex	Age (years)/Weight	Research content	sEVs isolation methods	sEV characterization methods
Senesi, 2024, Switzerland 16	Cells; Cross-sectional	STEMI	Ctrl (8); STEMI (8)	NR	≤ 85	Cargo + Intercellular communication	SEC + UC	NTA + TEM + FACS
Zhang, 2024, China 17	Cells; Cohort	STEMI undergoing PCI	T1 (98); T2 (97); T3 (99)	T1 (79.6); T2 (85.6); T3 (89.9)	T1 (62.46 ± 11.13); T2 (63.92 ± 11.86); T3 (60.99 ± 12.24)	Cargo + Intercellular communication	UC	NTA + TEM + WB
Osorio, 2024, Chile 18	Cells; Cross-sectional	①Myocardial ischemia; ②HF	①Ctrl (13); Non-Myocardial ischemia (13); Myocardial ischemia (13); ②Ctrl (10); CHF (10); DHF (10)	①Ctrl (NR); Non-Myocardial ischemia (84.62); Myocardial ischemia (53.84); ②Ctrl (NR); CHF (72.73); DHF (72.73)	①Ctrl (NR); Non-Myocardial ischemia (61 ± 12.86); Myocardial ischemia (64.92 ± 10.03); ②Ctrl (NR); CHF (65,27 ± 8,49); DHF (63.00 ± 17.54)	Cargo	UC/SEC	NTA + TEM + WB + Flow cytometry
Li, 2024, China^19^	Cells; Cross-sectional	Diabetic cardiomyopathy	Non-T2DM (30); T2DM (30)	Non-T2DM (50); T2DM (52.5)	Non-T2DM (48.77 ± 5.81); T2DM (51.13 ± 5.96)	Cargo + Intercellular communication	UC	NTA + TEM + WB
Zhou, 2023, China 20	Cells; Cross-sectional	AMI	Ctrl (4); AMI (5)	NR	NR	Detection methods for CM-sEVs	Quick exosome isolation kit + Peptide-anchored biomimetic interface	NTA + TEM + Flow cytometry + EIS + LSV
Abou, 2022, France 21	Cells; Cross-sectional	Ischemic HF	Non-cardiac cause of death patients (6); Heart transplant patients (6)	NR	NR	CM-sEVs specific marker	UC/Evtrap/PEG/PEG + UC/SEC	NTA + TEM + WB
Zhang, 2022, China 22	Cells; Cross-sectional	AMI	Ctrl (5); AMI (5)	Ctrl (60); AMI (60)	Ctrl (56.00 ± 10.44); AMI (51.20 ± 9.884)	Cargo + Intercellular communication	ExoQuick	NTA + TEM + WB
Hao, 2022, China 23	Cells; Cross-sectional	AF	Sinus Rhythm (50); AF (50)	Sinus Rhythm (64); AF (64)	Sinus Rhythm (60.70 ± 8.23); AF (62.26 ± 8.04)	Cargo + Intercellular communication	UC/ExoQuick	TEM + WB
Li, 2021, China 24	Cells; Cross-sectional	Chronic HF	Ctrl (5); Chronic HF (18)	Ctrl (60); Chronic HF (77)	Ctrl (60.4 ± 3.5); Chronic HF (61.58 ± 2)	Cargo + Intercellular communication	ExoQuick/ExoRNeasy/SEC	NTA + WB + DotBlot
Anselmo, 2021, Italy 25	Cells; Cross-sectional; Cohort	AS; Severe symptomatic AS; SIHD; ACS; HCM	Ctrl (52); AS (312); Severe symptomatic AS (8); SIHD (64); ACS (55); HCM (15)	Ctrl (57.7); AS (44.6); Severe symptomatic AS (50); SIHD (76.5); ACS (67.3); HCM (80)	Ctrl (61.3 ± 19.7); AS = 81.78 (76.90—86.34);Severe symptomatic AS = 79.87 (73—86);SIHD (69.46 ± 8.34);ACS (66.94 ± 11.74);HCM (48.48 ± 13)	Cargo + Intercellular communication + Detection methods for CM-sEVs	UC	NTA + WB + Cryo-electron microscopy + Immuno-gold TEM
Lin, 2021, China 26	Cells; Cross-sectional	AMI	Ctrl (45); AMI (43)	Ctrl (69); AMI (91)	Ctrl (60.45 ± 11.72); AMI (64.93 ± 13.77)	Cargo + Intercellular communication	ExoQuick	DLS + TEM + WB
Martins-Marques, 2020, Portugal 27	Cells; Cross-sectional	STEMI	Ctrl (29); STEMI (28)	Ctrl (82.1); STEMI (62.1)	Ctrl = 64 (53—67); STEMI = 65 (57—73)	Cargo	UC/Total Exosome Isolation Reagent/SEC	NTA + TEM + WB
Geng, 2020, China 28	Cells; Cross-sectional	MI	Ctrl (10); MI (14)	Ctrl (50); MI (57)	Ctrl (60.1 ± 5.2); MI (62.4 ± 4.5)	Cargo + Intercellular communication	UC	TEM + WB
Almeida, 2019, Portugal 29	Cells; Cross-sectional	AMI	Ctrl (12); AMI (15)	Ctrl (NR); AMI (60)	Ctrl (57.3 ± 18.4); AMI (65.5 ± 11.9)	Intercellular communication	UC	NTA + TEM + WB
Li, 2018, China 30	Cells; Cross-sectional	Myocardial ischemia	Ctrl (3); Ischemic (3)	NR	NR	Cargo + Intercellular communication	UC	NTA + TEM + WB
Yang, 2016, China 31	Cells; Cross-sectional	AMI	Ctrl (24); UAP (20); AMI 4h (26); AMI 24h (28); AMI 72h (25); AMI 7d (22)	Ctrl (58); UAP (55); AMI 4h (69); AMI 24h (57); AMI 72h (60); AMI 7d (59)	Ctrl (59 ± 13); UAP (54 ± 8); AMI 4h (59 ± 11); AMI 24h (57 ± 9); AMI 72h (59 ± 17); AMI 7d (60 ± 13)	Cargo + Intercellular communication	ExoQuick	TEM + WB + FACS
Li, 2025, America 32	Cells; Rats; Mice	Chronic HF (MI 6w)	①Sham; Model; ②Sham; Model	①Male; ②NR	①180—200g; ②NR	Cargo + Intercellular communication	UC	NTA + TEM + WB
Li, 2025, China 33	Cells; Rats	MA dependence	Ctrl; MA	Male	190-210g (2 months old)	Cargo + Intercellular communication	UC	NTA + TEM
Li, 2024, China 34	Cells; Mice	AMI (permanent LAD ligation)	Sham; AMI	NR	8—10 weeks old	Cargo + Intercellular communication	UC	TEM + WB
Zhang, 2024, China 35	Cells; Mice	DOX-induced cardiotoxicity	Ctrl; Dox	Male	7 weeks old	Intercellular communication	UC	NTA + TEM + WB + IF
Wang, 2024, China 36	Cells; Mice	HF (Ang II-treated)	Ctrl; Ang II	Male	8 weeks old	Cargo + Intercellular communication	UC	NTA + TEM + WB
Liu, 2024, China 37	Cells; Mice	I/R (LAD ligation for 45 min)	Sham; Sham+cEVs; I/R; I/R+cEVs	Male	6—8 weeks old	Cargo	UC	NTA + TEM + LSM + WB
Zhang, 2023, China 38	Cells; Mice	I/R (LAD ligation for 30 min)	Sham; MI/R	Male	6—8 weeks old	Intercellular communication + CM-sEVs specific marker	UC	NTA + TEM + WB + Flow cytometry
Schoger, 2023, Germany 39	Cells; Mice	①Cardiac remodeling/ hypertrophy (β-cat^Δex3^);②Early compensatory hypertrophy + late failing hypertrophy (TAC)	①Ctrl; β-cat^Δex3^;②Sham; TAC 5d; TAC 9w	Male / Female	①NR; ②17.5 months	Cargo	UC/MACS	NTA + TEM + WB + Fluorescent labeling
Liu, 2023, China 40	Cells; Canine	AF (Rapid atrial pacing model)	Sham; Pacing	NR	NR	Intercellular communication	UC	TEM + WB + IHC
Abou, 2022, France 21	Cells; Rats	MI (permanent LAD ligation	NR	NR	NR	CM-sEVs specific marker	UC/Evtrap/PEG/PEG + UC/SEC	NTA + TEM + WB
Gan, 2022, China 41	Cells; Mice	I/R (LAD ligation for 30 min)	Sham; I/R	Male	6—8 weeks old	Cargo+ Intercellular communication	UC	NTA + WB
Han, 2022, America 42	Mice	MI (permanent LAD ligation)	Sham; MI	Male	8—10 weeks old	Cargo + Intercellular communication	UC	NTA + TEM + WB
Li, 2021, China 24	Cells; Mice	Chronic ischemic HF (I/R 4w)	Sham; I/R 6-24h; I/R 4w	NR	8—14 weeks old	Cargo + Intercellular communication	ExoQuick/ExoRNeasy/SEC	NTA + WB + DotBlot
Anselmo, 2021, Italy 25	Cells; Porcine	Pressure overload porcine model (TAC)	TAC 56d	NR	NR	Cargo + Intercellular communication + Detection methods for CM-sEVs	UC	NTA + WB + Cryo-electron microscopy + Immuno-gold TEM
Martins-Marques, 2020, Portugal 27	Cells; Mice; Rats	①I/R (LAD ligation for 60 min);②Langendorff heart perfusion model	①Sham; I/R 30min; I/R 4h;②Ctrl; Ischemic	①Female;②NR	①10—12 weeks old;②10 weeks old	Cargo	UC/Total Exosome Isolation Reagent/SEC	NTA + TEM + WB
Zhang, 2020, China 43	Cells; Rats	AMI (permanent LAD ligation)	Sham; AMI; AMI+EGCG	Male	150—200g	Cargo	NR	NTA + TEM + WB
Chen, 2020, China 44	Cells; Rats	I/R (LAD ligation for 45min)	Sham; I/R; I/R + TXL	Male	220—250g	Cargo+ Intercellular communication	UC	NTA + TEM + WB
Vaskova, 2020, America 45	Cells; Rats	MI (permanent LAD ligation)	Sham; MI	Female	6—8 weeks old	Cargo	PEG precipitation	NTA
Luo, 2020, America 46	Cells; Mice	Tamoxifen-inducible cardiomyocyte CD63-NanoLuc mice	Tamoxifen-induced; Vehicle ctrl; Wild-type ctrl	NR	8—10 weeks old	Tracking of CM-sEVs	Exoquick/UC	NTA + TEM + WB
Rodriguez, 2018, Spain 47	Cells; Mice	MI (permanent LAD ligation)	Sham; MI	Male	12 weeks old	Intercellular communication	UC	NTA + DLS + FACS
Wang, 2016, America 48	Cells; Mice	Diabetic cardiomyopathy	Ctrl+STZ; HSP20TG+STZ	Male	6—8 weeks old	Cargo + Intercellular communication	UC	WB + DLS
Pironti, 2015, America 49	Mice	Cardiac pressure overload (TAC)	Sham; TAC	Male/Female	8—12 weeks old	Cargo	UC	NTA + TEM + WB

Abbreviations: ACS: Acute Coronary Syndrome; AF: Atrial Fibrillation; AMI: Acute Myocardial Infarction; Ang II: Angiotensin II; AS: Aortic Stenosis; cEVs: Cardiac-derived Extracellular Vesicles; CHF: Compensated Heart Failure; CM-sEVs: Cardiomyocyte-derived Small Extracellular Vesicles; CMUPRT: Cardiomyocyte-specific Uracil Phosphoribosyltransferase; Ctrl: Control; DHF: Decompensated Heart Failure; DLS: Dynamic Light Scattering; DOX: Doxorubicin; ECCG: Epigallocatechin Gallate; EIS: Electrochemical Impedance Spectroscopy; Evtrap: Extracellular Vesicles Total Recovery And Purification; FACS: Fluorescence-activated Cell Sorting; HCM: Hypertrophic Cardiomyopathy; HF: Heart Failure; HSP20TG: Heat Shock Protein 20 Transgenic; I/R: ischemia/reperfusion; IF: Immunofluorescence; IHC: Immunohistochemistry; LAD: Left Anterior Descending; LSM: Light Scattering Microscopy; LSV: Linear Sweep Voltammetry; MA: methamphetamine; MACS: Magnetic Activated Cell Sorting; MI: Myocardial Infarction; NR: Not Reported; NTA: Nanoparticle Tracking Analysis; PCI: Percutaneous Coronary Intervention; PEG: Polyethylene Glycol; SEC: Size Exclusion Chromatography; SIHD: Stable Ischemic Heart Disease; STEMI: ST-Elevation Myocardial Infarction; STZ: Streptozotocin; T2DM: Type 2 Diabetes Mellitus; T1 low: Patients divided into low miR-9-5p level; T2 medium: Patients divided into medium miR-9-5p level; T3 high: Patients divided into high miR-9-5p level; TEM: Transmission Electron Microscopy; TXL: Tongxinluo; UAP: Unstable Angina Pectoris; UC: Ultracentrifugation; WB: Western blotting; β-cat^Δex3^: Inducible cardiomyocyte-specific β-catenin gain-of-function mice

**Table 2:** Validation methods for cardiomyocyte-derived extracellular vesicles

Author, year	Cardiomyocyte type	Cells or tissues comparison	Tracking methods	Potential markers	Other validation methods
Li, 2025 32	Primary CMs	Primary CFs (sEVs-derived miR-21-5p levels)	Myh6-Cre/Rosa-mT/mG reporter mice	miR-208a (*in vivo*)	NR
Li, 2025 33	Primary CMs	NR	PKH26 (*in vitro*), PKH67 (*in vivo*)	NR	NR
Senesi, 2024 16	hiPSC-CMs	NR	DiR (*in vitro*)	CD172a (*in vivo*)	GW4869 (*in vitro*)
Zhang, 2024 17	Primary CMs	Primary CFs (effects of sEVs on neutrophil polarization)	PKH26 (*in vitro*), DiR (*in vivo*)	NR	GW4869 (*in vitro* and *in vivo*)
Li, 2024 34	HL-1	NR	Vector pCT-CD63-GFP (*in vitro*) , PKH67 (*in vitro*)	NR	NR
Zhang, 2024 35	H9c2	NR	DiO (*in vitro*)	Sarcomeric α-actinin (*in vivo*)	GW4869 (*in vitro* and *in vivo*)
Wang, 2024 36	Primary CMs	NR	DiO (*in vitro*)	NR	DMA (*vitro* and *vivo*)
Osorio, 2024 18	Primary CMs, AC16	NR	NR	NR	NR
Li, 2024 19	Primary CMs	Primary CFs (sEVs-derived miR-194-3p levels)	PKH67 (*in vitro*)	α-SA (*in vivo*)	NR
Liu, 2024 37	NR	Ecs, CFs, Smooth muscle cells, Macrophages (abundance of cell-specific marker RNAs in sEVs)	DiR (*in vitro*), Dil (*in vitro*)	Tnnt2, Myh6 (*in vivo*)	NR
Zhou, 2023 20	H9c2	NR	NR	CD172a (*in vitro* and *in vivo*)	Peptide-anchored biomimetic interface + EIS + LSV (*in vitro* and *in vivo*)
Zhang, 2023 38	Primary CMs	Primary CFs (number of sEVs and sEV-derived Ambra1 levels under H/R); Counts of Ambra1^+^ sEVs from left ventricles to aortic arch and peripheral blood circulation	PKH67 (*in vitro* and *in vivo*)	Ambra1 (*in vitro* and *in vivo*), sarcomeric α-actinin, MYH, cTnT (*in vivo*)	AAV9-cTnT-GFP-shAmbra1
Schoger, 2023 39	hiPSC-CMs	NR	NR	NR	NR
Liu, 2023 40	HL-1	NR	NR	cTnI (*in vivo*)	NR
Zhang, 2022 22	H9c2	NR	PKH26 (*in vitro*)	NR	NR
Hao, 2022 23	Primary CMs	NR	PKH67 (*in vitro*)	NR	NR
Abou, 2022 21	Primary CMs, H9c2	Primary CFs (sEVs-derived Ldb3 levels); Ldb3 expression was compared across tissues such as lung, liver, kidney, heart, plasma, leg muscle, brain, and skin	NR	Ldb3 (*in vivo*)	NR
Gan, 2022 41	Primary CMs	NR	AAV9-cTnT-CD63-GFP	NR	GW4869 (*in vivo*)
Han, 2022 42	NR	The metabolic labeling of tissue-specific miRNAs was compared among cardiomyocytes, skeletal muscle, kidney, hepatocytes, lung, spleen, and brain	Metabolic labeling of sEVs miRNAs	miR-208a, miR-1, α-MHC (*in vivo*)	NR
Li, 2021 24	Primary CMs	NR	Molecular beacons (*in vitro*), αMHC-MerCreMer-Rosa-mTmG reporter mice	Troponin T (*in vivo*)	NR
Anselmo, 2021 25	hiPSC-CMs	①Erythroid, Ecs, Platelet, Leucocyte, Monocyte (CD172a+ sEVs); ②Comparison of CM-EVs counts in coronary sinus, aortic arch, and peripheral blood	NR	CD172a (*in vitro* and *in vivo*), cTnT, sarcomeric α-actinin, miR-1, miR-133a (*in vivo*)	NR
Lin, 2021 26	AC16	NR	PKH67 (*in vitro*)	NR	NR
Martins-Marques , 2020 27	HL-1, H9c2	ECs (sEVs-derived Cx43 levels)	NR	Troponin T (*in vivo*)	NR
Geng, 2020 28	Primary CMs	Ecs, CFs (expression of sEVs markers)	PKH26 (*in vitro*)	NR	NR
Zhang, 2020 43	H9c2	NR	NR	NR	NR
Chen, 2020 44	Primary CMs	Primary CFs (sEVs effects on CMECs survival under H/R)	PKH26 (*in vitro*)	NR	GW4869 (*vitro* and *vivo*)
Vaskova, 2020 45	hiPSC-CMs	NR	NR	NR	NR
Luo, 2020 46	Primary CMs	NR	TG-αMHC-STOP-CD63 NanoLuc mice	NR	NR
Almeida, 2019 29	Primary CMs, H9c2	NR	PKH26 (*in vitro*)	Troponin T (*in vivo*)	NR
Li, 2018 30	Primary CMs	Ecs, CFs (expression of sEVs markers)	Calcein AM (*in vitro*)	NR	NR
Rodriguez, 2018 47	HL-1	NR	CFSE (*in vitro*)	Cx43 (*in vivo*)	NR
Yang, 2016 31	H9c2	NR	Vector pCT-CD63-GFP (*in vitro*), PKH67 (*in vitro*)	NR	NR
Wang, 2016 48	Primary CMs	NR	PKH67 (*in vitro*), DiR (*in vivo*)	NR	GW4869 (*in vivo*)
Pironti, 2015 49	NR	NR	NR	NR	①Cardiomyocyte- specific β-arrestin2 knockout in combination with pathological stimuli; ②DMA (*in vivo*)

Abbreviations: Ambra1: Activating molecule in BECN1-regulated autophagy protein 1; CD172a: Tyrosine-protein phosphatase non-receptor type substrate 1; CFs: Cardiac fibroblasts; CFSE: carboxyfluorescein succinimidyl amino ester; cTnI: cardiac troponin I; CMs: Cardiomyocytes; CM-sEVs: cardiomyocyte-derived small extracellular vesicles; cTnT: cardiac troponin T; Cx43: Connexin 43; DMA: dimethyl amiloride; ECs: Endothelial cells; EIS: Electrochemical Impedance Spectroscopy; H/R: hypoxia/reoxygenation; hiPSC-CMs: human induced pluripotent stem cell-derived cardiomyocytes; Ldb3: LIM Domain Binding 3; LSV: Linear Sweep Voltammetry; MHC: myosin heavy chain; Myh6: myosin heavy chain 6; NR: Not Reported; shAmbra1: short hairpin of Ambra1; Tnnt2: Troponin T type 2; α-SA: α-sarcomeric actin

**Table 3:** Screening and Applications of Potential Origin-tracing Markers for the CM-sEVs

Author, year	Potential markers (localization)	Sample sources	Marker Screening methods	Markers detection methods	Changes in sEVs markers across diseases	Results
Li, 2025 32	miR-208a	SD rats left ventricles (Chronic HF)	Known	qRT-PCR	miR-208a↑	Cardiac-specific miRNA-208a levels were increased in the sEVs derived from HF left ventricles compared to those derived from sham left ventricles.
Senesi, 2024 16	CD172a	hiPSC-CMs (normal)	Known	Flow cytometry	Present in sEVs	The sEVs expressed typical markers (CD9/CD63/CD81) and cardiomyocyte-specific markers (CD172a) to support their hiPSC-CMs origin.
Zhang, 2024 35	Sarcomeric α-actinin	C57BL/6J mice heart tissue (DOX)	Known	IF	Present in sEVs	DOX-treatment increased the expression of sEVs-related markers, CD63 and TSG101, in the sarcomeric α-actinin positive cardiomyocytes, thereby suggesting enhanced sEVs production or release from cardiomyocytes under DOX stress.
Liu, 2024 37	Tnnt2, Myh6	C57BL/6N mice heart tissue (normal)	Known	RT-qPCR	Present in sEVs	Higher expression of cardiomyocyte markers Tnnt2 and Myh6 in the sEVs than the markers for ECs, CFs, and macrophages indicates their cardiomyocyte origin.
Li, 2024 19	α-SA	Primary CMs (HG/HL)	Known	WB	Present in sEVs	The cardiomyocyte protein α-SA was detected in the sEVs fraction.
Zhang, 2023 38	Ambra1 (membrane surface), sarcomeric α-actinin, MYH, cTnT	①Primary neonatal mice CMs (H/R);②C57BL/6 mice left ventricular serum (MI/R)	Intersection analysis; Functional enrichment; Network screening; Structural prediction; Cellular expression analysis; Vesicles marker tracking; *In vivo* distribution	Flow cytometry	①Ambra1↑;②Ambra1↑	Ambra1 was used as a potential specific surface marker to identify CM-sEVs. Ambra1⁺ sEVs were enriched in cardiac-specific proteins such as sarcomeric α-actinin, MYH, and cTnT, further confirming their cardiomyocyte origin.
Liu, 2023 40	cTnI	Canine atrial tissue (Rapid-Pacing)	Known	IF	Present in sEVs	Immunofluorescence experiments showed co-localization of CD81 with the atrial cardiomyocyte marker cTnI.
Zhou, 2023 20	CD172a(membrane surface)	①H9c2 (normal); ②Patients serum (AMI)	Known	EIS + LSV	①Present in sEVs;②CD172a↑	CM-EVs were detected using a peptide-anchored biomimetic electrochemical interface targeting CD172a and showed high specificity, a wide linear range (10³-10⁸ particles/mL), a low detection limit (132 particles/mL), and reliable performance in the serum.
Abou, 2022 21	Ldb3	Patients left ventricles (Ischemic HF)	Differential proteomic analysis of sEVs from cells, heart tissue, and plasma; Verification of tissue-specific expression; Identification of cell-of-origin; Cross-validation using multiple sEVs isolation methods	WB	Ldb3 showed no significant change	Ldb3 was a potential specific marker of CM-sEVs and stably expressed in sEVs derived from the left ventricles of patients with ischemic HF.
Han, 2022 42	miR-208a, miR-1, α-MHC	CMUPRT double-transgenic mice peripheral blood (MI)	Known	RT-qPCR	miR-208↑, miR-1↑, α-MHC↑	MI mice peripheral blood sEVs showed increased levels of cardiomyocyte-specific miRNAs (miR-208a and miR-1) and the protein α-MHC.
Li, 2021 24	Troponin T	αMHC-MerCreMer-Rosa-mTmG reporter mice plasma (normal)	Known	WB	Present in sEVs	The sEVs fractions from plasma express cardiac Troponin T, along with GFP and Cre recombinase, and typical markers Alix and CD9, thereby forming their cardiomyocyte origin.
Anselmo, 2021 25	CD172a (membrane surface), cTnT, sarcomeric actinin, miR-1, miR-133a	①hiPSC-CMs (normal);②Human plasma (NR)	Multicolour flow cytometry-based method with a back-gating strategy; Detection of cardiomyocyte-specific proteins or miRNAs in sEVs contents; *In vivo* distribution	Multicolour flow cytometry-based method with a back-gating strategy	①AS:CD172a↑; one-year post-TAVR: CD172a↓;②Severe symptomatic AS after TAVR 2 months: CD172a↓;③SIHD: CD172a↑;④ACS: CD172a↑;⑤HCM: CD172a↑	CD172a was a putative surface marker for identifying CM-EVs, and CD172a⁺ EVs were enriched in cardiomyocyte-specific proteins such as cTnT and sarcomeric actinin, as well as heart-enriched microRNAs including miR-1 and miR-133a, suggesting their cardiomyocyte origin.
Martins-Marques, 2020 27	Troponin T	①Balb/c mice cardiac tissue (I/R ); ②Patients serum and plasma (STEMI)	Known	WB	Present in sEVs	The isolated sEVs were positive for troponin T, indicating that at least a portion of these sEVs originated from cardiomyocytes.
Almeida, 2019 29	Troponin T	Patients serum (AMI)	Known	WB	Present in sEVs	Serum-derived vesicles were positive for troponin T, suggesting that at least a subset of these sEVs were of cardiac origin.
Rodriguez, 2018 47	Cx43	C57BL/6J mice plasma (MI)	Known	Flow cytometry	MI 15d Cx43↑	Cx43 was regarded as a cardiomyocyte-enriched marker and can be incorporated into sEVs.

Abbreviations: ACS: Acute Coronary Syndrome; Ambra1: Activating molecule in BECN1-regulated autophagy protein 1; AMI: Acute Myocardial Infarction; AS: Aortic Stenosis; CD172a: Tyrosine-protein phosphatase non-receptor type substrate 1; CMs: Cardiomyocytes; CM-sEVs: Cardiomyocyte-derived Small Extracellular Vesicles; CMUPRT: Cardiomyocyte-specific Uracil Phosphoribosyltransferase; cTnI: cardiac troponin I; cTnT: cardiac troponin T; Cx43: Connexin 43; EIS: Electrochemical Impedance Spectroscopy; HCM: Hypertrophic Cardiomyopathy; HF: heart failure; HG/HL: high glucose/high lipid; hiPSC-CMs: human induced pluripotent stem cell-derived cardiomyocytes; H/R: Hypoxia/Reoxygenation; IF: Immunofluorescence; I/R: ischemia/reperfusion; Ldb3: LIM Domain Binding 3; LSV: Linear Sweep Voltammetry; MI: Myocardial Infarction; MI/R: Myocardial ischemia/reperfusion; MHC: myosin heavy chain; Myh6: myosin heavy chain 6; NG/NL: normal glucose/normal lipid; NR: Not Reported; SIHD: Stable Ischemic Heart Disease; STEMI: ST-Elevation Myocardial Infarction; TAVR: Transcatheter Aortic Valve Replacement; Tnnt2: Troponin T type 2; WB: Western blotting; α-SA: Alpha-sarcomeric actin; 4TUd: 4-Thiouridine

**Table 4:** Differential Changes in the Quantity and Cargos of CM-sEVs in Ischemic Heart Disease

Author,year	Species/ Strain	Diseases/Models (Methods)	Sample source	Quantity of sEVs relative to the control group	Cargos or biomarkers in sEVs relative to the control group	Results
Senesi, 2024 16	Human	STEMI	Serum	NR	miR-24-3p↓	Circulating sEVs from STEMI patients within 3 h of symptom onset (before PCI) showed decreased miR-24-3p levels compared with healthy controls.
Osorio, 2024 18	Human	Cardiac ischemia	Plasma	↑	hERG1↑, Hsp47↑	Compared to healthy subjects and ischemia-negative subjects, patients with positive cardiac ischemia showed elevated plasma levels of hERG1-sEVs and Hsp47-sEVs.
Li, 2024 33	C57BL/6 mice	AMI (permanent LAD ligation)	Serum	↑	MI 2-4h miR-30a↑, 72h return to normal	CM-sEVs-derived miR-30a aggravated post-AMI cardiac injury by inhibiting autophagy, inducing apoptosis, and promoting M1 macrophage polarization.
Zhou, 2023 20	Human	AMI	Serum	↑	NR	The peak LSV currents detected in serum samples from AMI patients were significantly higher than those from the control group.
Zhang, 2022 22	Human	AMI	Plasma	NR	HIF-1α↑	The expression levels of HIF-1α were higher in the hAMI-Exo compared with the hNor-Exo.
Han, 2022 42	CMUPRT double-transgenic mice	MI (permanent LAD ligation)	cardiac tissue/lung tissue	↑	miR-208 in cardiac tissue: MI 12h↑, MI 48h (return to baseline); miR-208 in lung tissue: MI 24h↑	Myocardial injury modulated lung gene expression and CM-sEVs-derived miR-208 potentially contributed to MI-associated pulmonary complications.
Anselmo, 2021 25	Human	SIHD, ACS	Plasma	CD172a^+^ EVs↑	CD172a↑	Compared with the control group, CD172a^+^ EVs were significantly increased in patients with SIHD or ACS.
Lin, 2021 26	Human	AMI	Serum	NR	lncRNA HCG15↑	The expression of lncRNA HCG15 positively correlated with cTnT levels and demonstrated clinical diagnostic value.
Martins-Marques, 2020 27	Human	STEMI	Serum, Plasma	↑	Cx43↓	The levels of circulating sEVs-derived Cx43 were significantly reduced in STEMI patients compared to the controls, indicating its potential as a plasma/serum biomarker of AMI.
Geng, 2020 28	Human	MI	Serum	NR	miR-143↓	The levels of serum sEVs-derived miR-143 were significantly decreased in patients with MI compared with controls, suggesting its potential as a biomarker for MI.
Zhang, 2020 43	SD rats	AMI (permanent LAD ligation)	Serum	↑	miR-30a↑	Upregulation of sEVs-derived miR-30a alleviated cardiac damage following AMI by suppressing apoptosis and inducing autophagy.
Vaskova,2020 45	SD rats	MI (permanent LAD ligation)	Plasma	↑	miR-181a↑	After MI, upregulation of miR-181a in CM-sEVs exacerbated myocardial fibrosis and hypertrophy.
Almeida,2019 29	Human	AMI	Serum	↑	NR	The number of sEVs were higher in AMI patients than in the controls.
Rodriguez, 2018 47	C57BL/6J mice	MI (permanent LAD ligation)	Plasma	↑	MI 15d Cx43↑	At early stages after infarction, CM-sEVs increased by 35%, but the differences only achieved statistical significance 15 days post-ischemia.
Li, 2018 30	Human	Myocardial Ischemia	Serum	NR	miR-939-5p↓	The levels of serum sEVs-derived miR-939-5p were significantly decreased in patients with ischemic compared with non-ischemic patients, suggesting its potential as a biomarker for myocardial ischemia.
Yang, 2016 31	Human	AMI	Serum	NR	UAP, AMI4h, AMI24h: miR-30a↑, 7d return to baseline	Serum miR-30a levels in AMI patients increased in a time-dependent manner; they increased markedly at 4 h, peaked at 24 h, and returned to baseline on day 7.

Abbreviations: ACS: Acute Coronary Syndrome; AMI: Acute Myocardial Infarction; CD172a: Tyrosine-protein phosphatase non-receptor type substrate 1; CM-sEVs: Cardiomyocyte-derived Small Extracellular Vesicles; CMUPRT: Cardiomyocyte-specific Uracil Phosphoribosyltransferase; Cx43: Connexin 43; Exo: Exosome; hAMI: acute myocardial infarction patients; HCG15: HLA complex group 15; hERG1: human ether-a-go-go-related gene 1; HIF-1α: hypoxia-inducible factor-1 alpha; hNor: human healthy controls; Hsp47: heat shock protein 47; LAD: Left Anterior Descending; LSV: linear sweep voltammetry; MI: Myocardial Infarction; NR: Not Reported; SIHD: Stable Ischemic Heart Disease; STEMI: ST-Elevation Myocardial Infarction; UAP: Unstable Angina Pectoris

**Table 5:** Differential Changes in the Quantity and Cargos of CM-sEVs in Myocardial Ischemia/Reperfusion Injury

Author, year	Species/ Strain	Diseases/Models (Methods)	Sample source	Quantity of sEVs relative to the control group	Cargos or biomarkers in sEVs relative to the control group	Results
Zhang, 2024 35	Human	STEMI undergoing PCI	Serum	NR	miR-9-5p↑	Elevated serum levels of sEVs-miR-9-5p independently predicted cardiovascular death in STEMI patients after PCI (AUC = 0.67).
Liu, 2024 37	C57BL/6N mice	I/R (LAD ligation for 45min)	cardiac tissue	NR	ATP5a1 (normal)	ATP5a1 mitigated oxidative stress-induced ferroptosis and mitochondrial dysfunction, preserved cardiac function, and reduced remodeling after I/R injury.
Zhang, 2023 38	C57BL/6 mice	I/R (LAD ligation for 30min)	Serum (left ventricular blood)	Ambra1+sEVs↑	Ambra1↑, mtDNA↑	Ambra1 served as an active marker of secretory autophagy-related CM-sEVs. Ambra1⁺ sEVs mediated the intercellular transfer of mitochondrial components, thereby contributing to ischemia-induced cardiac fibrosis.
Gan, 2022 41	C57BL/6J mice	I/R (LAD ligation for 30min)	Plasma	↑	miR-23-27-24 Cluster↑	I/R caused significant adipocyte ER stress and endocrine dysfunction by releasing miR-23-27-24 cluster-enriched sEVs.
Li, 2021 24	αMHC-MerCreMer- Rosa-mTmG reporter mice	I/R 6-24h (LAD ligation for 30min)	Plasma	NR	6-24h miR-30d↑; 48h return to baseline	miR-30d was protective in the acute phase of post-ischemic remodeling, but its subsequent decline in cardiac tissue and sEVs during the chronic phase was associated with adverse remodeling in rodent models.
Martins-Marques, 2020 27	①Balb/c mice; ②Wistar rats	①I/R 30min-4h (LAD ligation for 60 min); ②Langendorff heart perfusion model	Serum, left ventricles	↑	I/R 30min Cx43↓; I/R 4h Cx43 normalized	I/R induced a rapid and transient depletion of Cx43 in CM-sEVs via ischemia-triggered ubiquitination and autophagy, but Cx43 levels recovered during reperfusion.
Chen, 2020 44	SD rats	I/R (LAD ligation for 45min)	Serum	↑	I/R + TXL: linc-ROR↑	TXL-treatment CM-sEVs attenuated MI/R injury by modulating the linc-ROR/miR-145-5p/p70s6k1/eNOS axis and promoting endothelial cell survival.

Abbreviations: Ambra1: Activating molecule in BECN1-regulated autophagy protein 1; CM-sEVs: Cardiomyocyte-derived Small Extracellular Vesicles; Cx43: Connexin 43; eNOS: endothelial nitric oxide synthase; ER: endoplasmic reticulum; linc-ROR: Long Intergenic Non-Protein Coding RNA, Regulator Of Reprogramming; I/R: ischemia/reperfusion; LAD: Left Anterior Descending; mtDNA: Mitochondrial DNA; NR: Not Reported; PCI: Percutaneous Coronary Intervention; STEMI: ST-Elevation Myocardial Infarction; TXL: Tongxinluo

**Table 6:** Differential Changes in the Quantity and Cargos of CM-sEVs in Valvular Heart Disease, Cardiomyopathy, and Cardiac Arrhythmias

Author, year	Species/Strain	Diseases/Models (Methods)	Sample source	Quantity of sEVs relative to the control group	Cargos or biomarkers in sEVs relative to the control group	Results
Zhang, 2024 35	C57BL/6 mice	DOX-induced cardiotoxicity	Cardiac tissue	↑	NR	DOX suppressed SIRT1 expression, leading to downregulation of ATP6V1A and impaired lysosomal acidification, which resulted in accumulation of multivesicular bodies and mitochondria-derived vesicles and excessive sEVs secretion, thereby contributing to myocardial injury.
Li, 2024 19	Human	Diabetic cardiomyopathy	Serum	↑	miR-194-3p↓	Serum-derived sEVs from diabetic patients contain lower levels of miR-194-3p than those from non-diabetic individuals.
Schoger,2023 39	C57BL/6N mice	①Cardiac remodeling / hypertrophy (β-cat^Δex3^); ②Early compensatory hypertrophy/late failing hypertrophy (TAC)	Cardiac tissue	Early compensatory hypertrophy (TAC 5d)↑;Late failing hypertrophy (TAC 9w)↓	①PQC proteins↑, Cryab↑, ubiquitinated proteins↑, 20S proteasome α/β↑, Z-disk proteins↑; ②TAC 5d Cryab↑; TAC 9w Cryab↓	Cardiomyocytes subjected to Wnt activation or pressure-overload stress exhibited increased secretion of sEVs enriched in misfolding-prone Z-disk proteins, proteasome components, and chaperone-associated proteins. This aberrant cargo loading was alleviated during advanced cardiac remodeling.
Liu, 2023 40	Canine	AF (Rapid atrial pacing model)	Atrial tissue	↑	NR	KCa3.1 promoted atrial remodeling and enhanced sEVs secretion during rapid atrial pacing by increasing intracellular calcium and activating the AKT/Rab27a pathway.
Hao, 2022 23	Human	AF	Serum	NR	miR-210-3p↑	miR-210-3p levels in serum sEVs were significantly elevated in patients with atrial fibrillation compared with controls.
Anselmo, 2021 25	Human	①AS;②Severe symptomatic AS;③HCM	Plasma	CD172a^+^ EVs①AS: ↑; one-year post-TAVR:↓; ②Severe symptomatic AS after TAVR 2 months: ↓; ③HCM:↑	①AS: CD172a↑, Ceramide↑; one-year post-TAVR: CD172a↓;②Severe symptomatic AS after TAVR 2 months: CD172a↓	In patients with AS, the highest counts of circulating cardiac CD172a⁺ EVs were associated with a more favorable prognosis after TAVR compared with those with lower counts.
Anselmo, 2021 25	Porcine	pTAC	Plasma	pTAC 56d: CD172a⁺ EVs↑	CD172a↑	Cardiac pressure overload triggered the release of CD172a⁺ EVs from the heart.
Wang, 2016 48	FVB/N mice	Diabetic cardiomyopathy (STZ injection)	Serum	↑	Hsp20↑, SOD1↑, survivin↑, p-Akt↑	Hsp20 promoted the secretion of CM-sEVs via Tsg101. Hsp20TG sEVs protected against diabetic cardiac remodeling.
Pironti, 2015 49	C57/BL6 mice	Cardiac pressure overload (TAC)	Serum	↑	AT1R↑	Cardiac pressure overload induced the release of sEVs containing functional AT1R from cardiomyocytes; β-arrestin 2 regulated AT1R packaging without affecting sEVs secretion.

Abbreviations: AF: Atrial fibrillation; AKT: Protein Kinase B; AS: Aortic Stenosis; AT1R: Angiotensin II Type 1 Receptor; CD172a: Tyrosine-protein phosphatase non-receptor type substrate 1; pTAC: percutaneous transverse aortic constriction; Cryab: α-Crystallin B; DOX: Doxorubicin; HCM: Hypertrophic Cardiomyopathy; Hsp20TG: Heat Shock Protein 20 Transgenic; NR: Not Reported; PQC: protein quality control; p-Akt: phosphorylated AKT; Rab27a: Ras-related protein Rab-27A; SOD1: Superoxide Dismutase 1; STZ: streptozotocin; TAC: Transverse Aortic Constriction; β-cat^Δex3^: Inducible cardiomyocyte-specific β-catenin gain-of-function mice; TAVR: Transcatheter Aortic Valve Replacement; Tsg101: Tumor Susceptibility Gene 101; SIRT1: silencing information regulator 1

**Table 7:** Differential Changes in the Quantity and Cargos of CM-sEVs in Heart Failure

Author, year	Species/ Strain	Diseases/Models (Methods)	Sample source	Quantity of sEVs relative to the control group	Cargos or biomarkers in sEVs relative to the control group	Results
Li, 2025 32	SD rats	Chronic HF (MI 6w)	Left ventricles	NR	miR-21-5p↑, miR-208a↑	Left ventricles of chronic HF rats showed higher miR-21-5p expression in both lEVs and sEVs, whereas miR-208a levels were increased only in HF sEVs compared with the sham group.
Osorio, 2024 18	Human	HF	Plasma	Compensated HF: ↑; Decompensated HF:↓	Compensated HF: hERG1↑, Hsp47↑; Decompensated HF: hERG1↓, Hsp47↓	Compared with healthy subjects, hERG1 and Hsp47 in plasma sEVs were increased in compensated HF but significantly decreased in decompensated HF.
Wang, 2024 36	C57BL/6 mice	HF (Ang II-treated)	NR	↑	Shh/N-Shh/Gli1↑	Ang II-induced cardiomyocyte injury led to cardiac hypertrophy and fibrosis through the release of sEVs enriched in Shh signaling components.
Abou, 2022 21	Human	Ischemic HF	Left ventricles	NR	Ldb3 showed no significant change	The expression of Ldb3 (78 kDa) in myocardial sEVs did not differ significantly between patients with ischemic HF and controls.
Li, 2021 24	Human	Chronic ischemic HF	Plasma	NR	miR-30d-5p↓	miR-30d levels in CM-sEVs from chronic HF patients were lower than in controls. In HF patients, higher sEV miR-30d levels were associated with better ventricular function, thereby linking reduced miR-30d levels to adverse cardiac remodeling.
Li, 2021 24	αMHC-MerCreMer-Rosa-mTmG reporter mice	Chronic ischemic HF (I/R 4w)	Plasma	NR	miR-30d↓	miR-30d protected against acute post-ischemic remodeling, but its decline in cardiac tissue and sEVs during the chronic phase contributed to adverse remodeling in rodent models.

Abbreviations: Ang II: Angiotensin II; CM-sEVs: Cardiomyocyte-derived small extracellular vesicles; Gli1: GLI family zinc finger 1; hERG1: human ether-a-go-go-related gene 1; HF: heart failure; Hsp47: heat shock protein 47; I/R: ischemia/reperfusion; Ldb3: LIM Domain Binding 3;lEVs: large extracellular vesicles; LVEF: Left Ventricular Ejection Fraction; MI: Myocardial Infarction; NR: Not Reported; Shh: Sonic hedgehog

**Table 8:** CM-sEVs-Mediated Communication Between Cardiomyocytes and the Underlying Mechanisms

Author, year	Source of sEVs	Treatment	Cargos	Target cells	Tracking methods	Downstream targets	Pathological mechanism	Molecular mechanism
Li, 2024 34	HL-1	Hypoxia	miR-30a↑	Normoxic HL-1	Vector pCT- CD63-GFP (*in vitro*), PKH67 (*in vitro*)	Beclin-1↓, ATG5↓	Autophagy↓, Apoptosis↑, Inflammation↑	miR-30a inhibited autophagy by targeting Beclin-1/ATG5, promoted cardiomyocyte apoptosis, and induced macrophage polarization toward a pro-inflammatory M1 state, thereby exacerbating myocardial injury.
Zhang, 2024 35	H9c2	DOX	NR	Normal H9c2	DiO (*in vitro*)	NR	Oxidative stress↑	The sEVs derived from DOX-treated cardiomyocytes promote oxidative stress in recipient cells by enhancing ROS production and impairing antioxidant defenses.
Wang, 2024 36	Primary CMs	Ang II-stimulated	Shh/N-Shh/Gli1↑	Normal CMs	DiO (*in vitro*)	NR	Cardiac hypertrophy↑	Ang II induced cardiac hypertrophy by stimulating the secretion of sEVs carrying Shh/N-Shh/Gli1, which activated the Shh signaling pathway.
Liu, 2024 37	Primary CMs	H/R + cEVs	ATP5a1 (normal)	Primary CMs	DiR (*in vitro*), Dil (*in vitro*)	NR	Oxidative stress↓, Cell Ferroptosis↓, Mitochondrial dysfunction↓	Healthy mouse-derived CM-sEVs delivered ATP5a1 to the H/R-treated cardiomyocytes, thereby reducing mitochondrial ROS, mitigating damage, and inhibiting ferroptosis.
Lin, 2021 26	AC16	Hypoxia	lncRNA HCG15↑	Normoxic AC16	PKH67 (*in vitro*)	NF-κB/p65↑, p38 MAPK↑	Apoptosis↑, Inflammation↑	Hypoxia-enriched lncRNA HCG15 in CM-sEVs activated the NF-κB/p65 and p38 MAPK pathways, thereby triggering apoptosis and inflammation in recipient cardiomyocytes and exacerbating acute ischemic injury.
Yang, 2016 31	H9c2	Hypoxia	miR-30a↑	Normoxic H9c2	Vector pCT-CD63-GFP (*in vitro*), PKH67 (*in vitro*)	Beclin-1↓, ATG12↓	Autophagy↓	MiR-30a was upregulated in sEVs from hypoxic cardiomyocytes. Inhibition of miR-30a or blockade of exosome release restored Beclin-1, Atg12, and the LC3II/LC3I ratio, thereby maintaining autophagy and providing cardioprotection.
Wang, 2016 48	Primary CMs	Hsp20TG + Hyperglycemia	Hsp20↑, SOD1↑, survivin↑, p-Akt↑	Primary CMs	PKH67 (*in vitro*)	NR	Oxidative stress↓	CM-sEVs from Hsp20TG mice were enriched with Hsp20, p-Akt, survivin, and SOD1 and attenuated high glucose-induced oxidative stress in cardiomyocytes.

Abbreviations: Ang II: Angiotensin II; ATG5: Autophagy Related 5; ATG12: Autophagy Related 12; cEVs: Cardiac-derived extracellular vesicles; CMs: car*diomyocytes; CM-sEVs: Cardiomyocyte-derived Small Extracellular Vesicles; DOX: Doxorubicin; H/R: Hypoxia/Reoxygenation; Gli1: GLI family zinc finger 1; Hsp20: Heat shock protein 20; Hsp20TG: Heat shock protein 20 transgenic; NF-κB: Nuclear Factor kappa-light-chain-enhancer of activated B cells; NR: Not Reported; p-Akt: Phosphorylated Protein kinase B; MAPK: Mitogen-Activated Protein Kinase; ROS: Reactive Oxygen Species; SOD1: Superoxide Dismutase 1*

**Table 9:** CM-sEVs-Mediated Communication Between Cardiomyocytes and Fibroblasts

Author, year	Source of sEVs	Treatment	Cargos	Target cells	Tracking methods	Downstream targets	Pathological mechanism	Molecular mechanism
Senesi, 2024 16	hiPSC-CMs	ischemia-like conditions	miR-24-3p↓	Human CFs	DiR (*in vitro*)	FURIN↑, CCND1↑, SMAD4↑	Fibrosis↑	Downregulation of miR-24-3p in CM-sEVs derepressed TGF-β1 signaling, thereby promoting cardiac fibroblast activation and fibrosis.
Li, 2024 19	Primary CMs	High glucose/high lipid	miR-194-3p↓	Primary CFs	PKH67 (*in vitro*)	TGFβR2↑	Fibrosis↑	Downregulation of miR-194-3p in CM-sEVs derepressed TGFβR2, thereby activating TGF-β/Smad signaling and promoting fibroblast activation and fibrosis.
Wang, 2024 36	Primary CMs	Ang II-stimulated	Shh/N-Shh/Gli1↑	Primary CFs	DiO (*in vitro*)	NR	Fibrosis↑	Ang II promoted cardiac fibroblast activation and proliferation by stimulating the secretion of sEVs enriched in Shh/N-Shh/Gli1, which in turn activated the Shh signaling pathway
Zhang, 2023 38	Primary CMs	H/R	mtDNA↑	Primary CFs	PKH67 (*in vitro*)	cGAS-STING↑	Fibrosis↑	Ambra1^+^ sEVs delivered mtDNA to fibroblasts and activated the cGAS-STING pathway, thereby promoting fibroblast proliferation.
Hao, 2022 23	Primary CMs	Tachypacing for 24h	miR-210-3p↑	Primary CFs	PKH67 (*in vitro*)	GPD1L↓	Atrial fibrosis↑	Elevated miR-210-3p in atrial CM-sEVs targeted GPD1L and promoted atrial fibrosis via the PI3K/AKT signaling pathway.
Li, 2021 24	Primary CMs	Hypoxia	miR-30d-5p↑	Primary CFs	Molecular beacons (*in vitro*), αMHC-MerCreMer-Rosa-mTmG reporter mice	Itga5↓	Fibrosis↓	Elevated CM-sEVs miR-30d-5p suppressed cardiac fibroblast proliferation and collagen deposition by inhibiting Itga5 and blocking the TGF-β/Smad pathway.

Abbreviations: Ambra1: Activating molecule in BECN1-regulated autophagy protein 1; AKT: Protein kinase B; Ang II: Angiotensin II; CCND1:Cyclin D1; CFs: cardiac fibroblasts; cGAS: cyclic GMP-AMP synthase; CMs: Cardiomyocytes; CM-sEVs: Small Extracellular Vesicles derived from Cardiomyocytes; Gli1:GLI family zinc finger 1; GPD1L:glycerol-3-phosphate dehydrogenase 1-like; hiPSC-CMs: human induced pluripotent stem cell derived cardiomyocytes H/R: Hypoxia/Reoxygenation; mtDNA: Mitochondrial DNA; NR: Not Reported; PI3K:Phosphoinositide 3-kinase; TGF-β1:transforming growth factor-β-1; TGFβR2:Transforming growth factor-β- receptor type II; STING: stimulator of interferon genes

**Table 10:** CM-sEVs-Mediated Communication Between Cardiomyocytes and Endothelial Cells

Author, year	Source of sEVs	Treatment	Cargos	Target cells	Tracking methods	Downstream targets	Pathological mechanism	Molecular mechanism
Geng, 2020 28	Primary CMs	MI	miR-143↓	HUVECs	PKH26 (*in vitro*)	IGF-IR↑	Angiogenesis↑	Downregulation of miR-143 promoted angiogenesis in endothelial cells by targeting IGF-IR and enhancing NO production.
Chen, 2020 44	Primary CMs	H/R + TXL	linc-ROR↑	CMECs	PKH26 (*in vitro*)	miR-145-5p↓	Oxidative stress↓, Apoptosis↓	TXL-induced CM-sEVs were enriched in linc-ROR, which suppressed miR-145-5p in CMECs, activated the eNOS/p70s6k1 pathway, and enhanced CMEC survival by inhibiting oxidative stress and apoptosis.
Rodriguez, 2018 47	HL-1	Normal	NR	Primary ECs, MS1 ECs	CFSE (*in vitro*)	MMP3↑	Cell migration↑, Cell proliferation↑, Apoptosis↑	CM-sEVs promoted endothelial cell migration and proliferation, and concurrently increased MMP3 secretion. Excessive MMP3 activity caused excessive extracellular matrix degradation, impaired tube formation, and endothelial cell death.
Li, 2018 30	Primary CMs	Hypoxia	miR-939-5p↓	HUVECs	Calcein AM (*in vitro*)	iNOS↑	Cell proliferation↑, Angiogenesis↑	Downregulation of miR-939-5p activated the iNOS-NO signaling pathway, leading to increased endothelial cell proliferation and enhanced angiogenesis.
Wang, 2016 48	Primary CMs	Hyperglycemia	Hsp20↑, SOD1↑, survivin↑, p-Akt↑	Primary ECs	PKH67 (*in vitro*)	NR	Oxidative stress↓	CM-sEVs from Hsp20TG mice were enriched with Hsp20, p-Akt, survivin, and SOD1, and attenuated high glucose-induced oxidative stress in endothelial cells.

Abbreviations: CFSE: carboxyfluorescein succinimidyl amino ester; CMECs: cardiac microvascular endothelial cells; CM-sEVs: Cardiomyocyte-derived Small Extracellular Vesicles; ECs: endothelial cells; eNOS: endothelial nitric oxide synthase; Hsp20: Heat shock protein 20; Hsp20TG: Heat shock protein 20 transgenic; HUVECs: Human Umbilical Vein Endothelial Cells; H/R: Hypoxia/Reoxygenation; IGF-IR: insulin-like growth factor 1 receptor; iNOS: Inducible Nitric Oxide Synthase; linc-ROR: Long Intergenic Non-Protein Coding RNA, Regulator Of Reprogramming; MMP3: matrix metalloproteinase 3; NF-κB: Nuclear Factor kappa-light-chain-enhancer of activated B cells; NLK: nemo-like kinase; NO: Nitric Oxide; NR: Not Reported; p-Akt: Phosphorylated Protein kinase B; SOD1: Superoxide Dismutase 1; TXL: Tongxinluo

**Table 11:** CM-sEVs-Mediated Communication Between Cardiomyocytes and Other Cell Types

Author, year	Source of sEVs	Treatment	Cargos	Target cells	Tracking methods	Downstream targets	Pathological mechanism	Molecular mechanism
Li, 2025 32	Myh6-Cre/Rosa-mT/mG reporter mice	Chronic HF (MI 6w)	miR-21-5p↑	Brain microglial cells	Myh6-Cre/Rosa-mT/mG reporter mice	PRMT1↓	Inflammation↑	During MI-induced HF, CM-sEVs with elevated miR-21-5p inhibited PRMT1 expression, promoting M1 polarization of brain microglia and neuroinflammation, thereby contributing to cardiogenic cognitive impairment.
Li, 2025 33	Primary CMs	MA-induced	miR-181a-5p↑	SH-SY5Y cell	PKH26 (*in vitro*)	HSP90B1, RAD21, MAP2K1, TNF, TIMP3, RAD23B (Dual-luciferase)	Steroid biosynthetic, Amide metabolic, Apoptosis (predicted)	CM-sEVs delivered miR-181a-5p, facilitating heart-brain crosstalk and aggravating MA-induced conditioned place preference behavior in rats. These effects were mediated through the regulation of key brain targets, including HSP90B1, TNF, and MAP2K1.
Zhang, 2024 17	Primary CMs	H/R	miR-9-5p↑	Bone marrow-derived neutrophils	PKH26 (*in vitro*), DiR (*in vivo*)	SOCS5↓, SIRT1↓	Inflammation↑	The levels of miR-9-5p in CM-sEVs were upregulated during H/R and promoted N1 neutrophil polarization via the SIRT1/NF-κB and SOCS5/JAK2/STAT3 pathways, thereby amplifying inflammatory responses.
Li, 2024 34	HL-1	Hypoxia	miR-30a↑	RAW264.7	Vector pCT-CD63-GFP (*in vitro*), PKH67 (*in vitro*)	Beclin-1↓, ATG5↓	Autophagy↓, Apoptosis↑, Inflammation↑	MiR-30a inhibited autophagy by targeting Beclin-1/ATG5, promoted cardiomyocyte apoptosis, and induced macrophage polarization toward a pro-inflammatory M1 phenotype, thereby exacerbating myocardial injury.
Liu, 2023 40	HL-1	Rapid pacing	NR	RAW264.7	NR	NR	Inflammation↑	KCa3.1 induced M1 macrophage polarization by promoting the release of pro-inflammatory sEVs via calcium signaling and AKT/Rab27a activation.
Zhang, 2022 22	H9c2	Hypoxia	HIF-1α↑, TGF-β↑	RAW264.7	PKH26 (*in vitro*)	NR	Oxidative stress↓	CM-sEVs induced M2 macrophage polarization via TGF-β/Smad3 signaling, reduced oxidative stress in H9c2 cardiomyocytes, and promoted cardiac repair.
Gan, 2022 41	MI/R mice	MI/R	miR-23-27-24 Cluster↑	3T3L1 adipocytes	AAV9-cTnT-CD63-GFP	EDEM3↓	ER stress↑, Endocrine dysfunction↑	During MI/R, adipocytes took up CM-sEVs enriched with the miR-23-27-24 cluster, which suppressed EDEM3, induced ER stress, and reduced adiponectin synthesis, thereby establishing a deleterious heart-adipose feedback loop.
Han, 2022 42	CMUPRT double-transgenic mice	MI	miR-208a↑	ECs (lung)	Cell type-specific metabolic labeling and tracking of sEVs miRNAs	NLK↓, Tmbim6↓	Inflammation↑	After MI, lung endothelial cells were targeted by CM-sEVs-derived miR-208a, which suppressed NLK and Tmbim6, leading to activation of NF-κB signaling and resulting in pulmonary inflammation and remodeling.
Almeida, 2019 29	H9c2, Primary CMs	Ischemia	NR	Peritoneal macrophages, RAW264.7	PKH26 (*in vitro*)	NR	Inflammation↓	In ischemic conditions, CM-sEVs modulated the p38 MAPK and NF-κB signaling pathways, which led to reduced macrophage inflammation, phagocytosis, and adhesion, thereby weakening their support for cardiomyocytes and impairing cardiac repair.

Abbreviations: AKT: Protein kinase B; ATG5: Autophagy Related 5; CM-sEVs: Cardiomyocyte-derived Small Extracellular Vesicles; EDEM3: ER degradation enhancing alpha-mannosidase like protein 3; ER: endoplasmic reticulum; HF: heart failure; HIF-1α: Hypoxia-Inducible Factor 1 α; H/R: Hypoxia/Reoxygenation; JAK2: Janus kinase 2; MA: methamphetamine; MI: Myocardial Infarction; MI/R: Myocardial Ischemia/Reperfusion; NF-κB: Nuclear Factor kappa-light-chain-enhancer of activated B cells; NR: Not Reported; PRMT1: protein arginine methyltransferase 1; Rab27a: Ras-related protein Rab-27A; SIRT1: silencing information regulator 1; SOCS5: suppressor of cytokine signaling 5; STAT3: signal transducer and activator of transcription 3; TGF-β: transforming growth factor-β
