# Supplementary material for: The Origin and Application of Cardiomyocyte-Derived Small Extracellular Vesicles: A Systematic Review
Source: Int J Med Sci. 2026 May 18;23(7):2252–76. doi: 10.7150/ijms.133199 (PMC13280738; doi:10.7150/ijms.133199)
Supplement: Supplementary file 1 — Supplementary tables. [file ijmsv23p2252s1.zip › Supplementary Material+Figure/Supplementary Material.pdf]

Supplementary Materials

Table S1: Search Terms

Databases	Search Terms	Number
**PubMed**	("extracellular vesicles"[MeSH Terms] OR "extracellular vesicle"[Title/Abstract] OR "vesicle extracellular"[Title/Abstract] OR "vesicles extracellular"[Title/Abstract] OR "Exosomes"[Title/Abstract] OR "Exosome"[Title/Abstract] OR "Exosomes"[MeSH Terms]) AND ("Heart"[MeSH Terms] OR "Hearts"[Title/Abstract] OR "myocardium"[MeSH Terms] OR "cardiac muscle"[Title/Abstract] OR "Myocardia"[Title/Abstract] OR "muscle cardiac"[Title/Abstract] OR "cardiac muscles"[Title/Abstract] OR "muscles cardiac"[Title/Abstract] OR "muscle heart"[Title/Abstract] OR "heart muscle"[Title/Abstract] OR "heart muscles"[Title/Abstract] OR "muscles heart"[Title/Abstract] OR "myocytes cardiac"[Title/Abstract] OR "myocytes, cardiac"[MeSH Terms] OR "cardiac myocyte"[Title/Abstract] OR "cardiac myocytes"[Title/Abstract] OR "myocyte cardiac"[Title/Abstract] OR "muscle cells cardiac"[Title/Abstract] OR "cardiac muscle cell"[Title/Abstract] OR "cardiac muscle cells"[Title/Abstract] OR "cell cardiac muscle"[Title/Abstract] OR "cells cardiac muscle"[Title/Abstract] OR "muscle cell cardiac"[Title/Abstract] OR "muscle cells heart"[Title/Abstract] OR "cell heart muscle"[Title/Abstract] OR "cells heart muscle"[Title/Abstract] OR "heart muscle cell"[Title/Abstract] OR "heart muscle cells"[Title/Abstract] OR "muscle cell heart"[Title/Abstract] OR "Cardiomyocytes"[Title/Abstract] OR "Cardiomyocyte"[Title/Abstract])	1193
**Web of Science**	(TS=(Extracellular Vesicles) OR TS=(Extracellular Vesicle) OR TS=(Vesicle, Extracellular) OR TS=(Vesicles, Extracellular) OR TS=(Exosomes) OR TS=(Exosome)) AND (TS=(Heart) OR TS=(Hearts) OR TS=(Myocardium) OR TS=(Cardiac Muscle) OR TS=(Myocardia) OR TS=(Muscle, Cardiac) OR TS=(Cardiac Muscles) OR TS=(Muscles, Cardiac) OR TS=(Muscle, Heart) OR TS=(Heart Muscle) OR TS=(Heart Muscles) OR TS=(Muscles, Heart) OR TS=(Myocytes, Cardiac) OR TS=(Cardiac Myocyte) OR TS=(Cardiac Myocytes) OR TS=(Myocyte, Cardiac) OR TS=(Muscle Cells, Cardiac) OR TS=(Cardiac Muscle Cell) OR TS=(Cardiac Muscle Cells) OR TS=(Cell, Cardiac Muscle) OR TS=(Cells, Cardiac Muscle) OR TS=(Muscle Cell, Cardiac) OR TS=(Muscle Cells, Heart) OR TS=(Cell, Heart Muscle) OR TS=(Cells, Heart Muscle) OR TS=(Heart Muscle Cell) OR TS=(Heart Muscle Cells) OR TS=(Muscle Cell, Heart) OR TS=(Cardiomyocytes) OR TS=(Cardiomyocyte))	2818

Databases	Search Terms	Number
**Cochrane**	(Extracellular Vesicles or Vesicles, Extracellular or Extracellular Vesicle or Vesicle, Extracellular or Exosomes or Exosome) and (Heart or Hearts or Myocardium or Heart Muscles or Myocardia or Muscles, Cardiac or Muscle, Cardiac or Cardiac Muscle or Muscles, Heart or Muscle, Heart or Cardiac Muscles or Heart Muscle or Myocytes, Cardiac or Cardiac Myocyte or Muscle Cell, Heart or Muscle Cell, Cardiac or Cardiac Muscle Cells or Heart Muscle Cells or Cells, Cardiac Muscle or Cardiomyocytes or Heart Muscle Cell or Cells, Heart Muscle or Cell, Cardiac Muscle or Myocyte, Cardiac or Muscle Cells, Cardiac or Cardiac Myocytes or Cardiomyocyte or Muscle Cells, Heart or Cell, Heart Muscle or Cardiac Muscle Cell)	23
**Embase**	('exosome'/exp OR 'exosome' OR 'exosomes'/exp OR 'exosomes' OR 'extracellular vesicle'/exp OR 'extracellular vesicle' OR 'extracellular vesicles'/exp OR 'extracellular vesicles') AND ('heart'/exp OR 'heart' OR 'cardiac effect'/exp OR'cardiac effect' OR 'cor'/exp OR 'cor' OR 'myocardial tissue'/exp OR 'myocardial tissue' OR 'heart muscle'/exp OR 'heart muscle' OR 'heart muscle structure'/exp OR 'heart muscle structure' OR 'heart muscle volume'/exp OR 'heart muscle volume' OR 'muscle, heart'/exp OR 'muscle, heart' OR 'myocardial muscle'/exp OR 'myocardial muscle' OR 'myocardial structure'/exp OR 'myocardial structure' OR 'myocardium'/exp OR 'myocardium' OR 'myocardium extract'/exp OR 'myocardium extract' OR 'myocardium structure'/exp OR 'myocardium structure' OR 'cardiac muscle'/exp OR 'cardiac muscle' OR 'cardiac cell'/exp OR 'cardiac cell' OR 'cardiac muscle cell fiber'/exp OR 'cardiac muscle cell fiber' OR 'cardiac muscle fiber'/exp OR 'cardiac muscle fiber' OR 'cardiac muscle fiber length'/exp OR 'cardiac muscle fiber length' OR 'cardiac myocyte'/exp OR 'cardiac myocyte' OR 'cardiac myocytes'/exp OR 'cardiac myocytes' OR'cell, myocardial'/exp OR 'cell, myocardial' OR 'heart cell'/exp OR 'heart cell' OR 'heart fiber'/exp OR 'heart fiber' OR 'heart muscle cell'/exp OR 'heart muscle cell' OR 'heart muscle cell fiber'/exp OR 'heart muscle cell fiber' OR 'heart muscle fiber'/exp OR 'heart muscle fiber' OR 'heart muscle fiber length'/exp OR 'heart muscle fiber length' OR 'heart muscle fibre'/exp OR 'heart muscle fibre' OR 'heart muscle fibre length'/exp OR 'heart muscle fibre length' OR 'muscle cell, heart'/exp OR 'muscle cell, heart' OR 'myocardial cell'/exp OR 'myocardial cell' OR 'myocardial cell fiber'/exp OR 'myocardial cell fiber' OR 'myocardial	3767

Databases	Search Terms	Number
	fiber'/exp OR 'myocardial fiber' OR 'myocardial fiber length'/exp OR 'myocardial fiber length' OR 'myocardial fibre length'/exp OR 'myocardial fibre length' OR 'myocardium cell'/exp OR 'myocardium cell' OR 'myocytes, cardiac'/exp OR 'myocytes, cardiac' OR 'cardiac muscle cell'/exp OR 'cardiac muscle cell')	

Table S2: Cross-Sectional studies Quality

Author, year	Q1	Q2	Q3	Q4	Q5	Q6	Q7	Q8	Q9	Q10	Q11	Total scores (11 points)	Quality grade
Senesi, 2024 [16]	1	1	0	0	0	1	1	0	0	1	0	5	moderate-quality
Osorio, 2024 [18]	1	1	0	0	0	1	1	1	1	1	0	7	moderate-quality
Li, 2024 [19]	1	1	1	0	0	1	0	1	0	1	0	6	moderate-quality
Zhou, 2023 [20]	1	0	0	0	0	1	1	0	0	1	0	4	moderate-quality
Abou, 2022 [21]	1	1	1	0	0	1	0	0	0	1	0	5	moderate-quality
Zhang, 2022 [22]	1	1	1	0	0	1	1	1	0	1	0	7	moderate-quality
Hao, 2022 [23]	1	1	1	0	0	1	1	0	0	1	0	6	moderate-quality
Li, 2021 [24]	1	0	0	0	0	1	1	0	1	1	0	5	moderate-quality
Anselmo, 2021 [25]	1	0	1	0	0	1	0	0	0	1	0	4	moderate-quality
Lin, 2021 [26]	1	0	1	0	0	1	0	0	0	1	0	4	moderate-quality
Martins-Marques , 2020 [27]	1	1	1	0	0	1	1	1	0	1	0	7	moderate-quality
Geng, 2020 [28]	1	1	1	0	0	1	1	1	0	1	0	7	moderate-quality
Almeida, 2019 [29]	1	0	0	0	0	1	0	0	0	1	0	3	low-quality

Li,2018 [30]	1	1	1	0	0	1	1	0	0	1	0	6	moderate-quality
Yang, 2016 [31]	1	1	1	0	0	1	1	0	0	1	0	6	moderate-quality

The total score is 11 points:0–3 deemed low-quality; 4–7 deemed moderate-quality;8–11 deemed high-quality.

Q1: Define the source of information (survey, record review);

Q2: List inclusion and exclusion criteria for exposed and unexposed subjects (cases and controls) or refer to previous publications;

Q3: Indicate time period used for identifying patients;

Q4: Indicate whether or not subjects were consecutive if not population-based;

Q5: Indicate if evaluators of subjective components of study were masked to other aspects of the status of the participants;

Q6: Describe any assessments undertaken for quality assurance purposes (e.g., test/retest of primary outcome measurements);

Q7: Explain any patient exclusions from analysis;

Q8: Describe how confounding was assessed and/or controlled;

Q9: If applicable, explain how missing data were handled in the analysis;

Q10: Summarize patient response rates and completeness of data collection;

Q11: Clarify what follow-up, if any, was expected and the percentage of patients for which incomplete data or follow-up was obtained.

Table S3: Cohort Studies Quality

Author,year	Q1	Q2	Q3	Q4	Q5	Q6	Q7	Q8	Total scores (9points)	Quality grade
Zhang, 2024 [17]	1	0	1	1	2	1	1	1	8	high-quality
Anselmo, 2021 [25]	1	0	1	1	2	1	1	1	8	high-quality
Q1: Representativeness of the exposed cohort;<br>Q2: Selection of the non exposed cohort;<br>Q3: Ascertainment of exposure;<br>Q4: Demonstration that outcome of interest was not present at start of study;<br>Q5: Comparability of cohorts on the basis of the design or analysis;<br>Q6: Assessment of outcome;<br>Q7: Was follow-up long enough for outcomes to occur;<br>Q8: Adequacy of follow up of cohorts.										

Table S4: SYRCLE's Risk of Bias Tool of Animal Experiments

Author, year	Sequence generation	Baseline characteristics	Allocation concealment	Random housing	Blinding (Performance bias)	Random outcome assessment	Blinding (Detection bias)	Incomplete outcome data	Selective outcome reporting	Other sources of bias
Li, 2025 [32]	unclear	unclear	unclear	unclear	unclear	unclear	unclear	low risk	low risk	unclear
Li, 2025 [33]	unclear	unclear	unclear	unclear	unclear	unclear	unclear	low risk	low risk	low risk
Li, 2024 [34]	unclear	low risk	unclear	unclear	unclear	unclear	unclear	unclear	low risk	unclear
Zhang, 2024 [35]	unclear	low risk	unclear	unclear	unclear	unclear	low risk	unclear	low risk	unclear
Wang, 2024 [36]	unclear	unclear	unclear	low risk	unclear	unclear	unclear	unclear	low risk	unclear
Liu, 2024 [37]	unclear	unclear	unclear	unclear	unclear	unclear	unclear	unclear	low risk	unclear
Zhang, 2023 [38]	unclear	unclear	unclear	unclear	unclear	unclear	unclear	unclear	low risk	unclear
Schoger,2023 [39]	unclear	unclear	unclear	unclear	low risk	unclear	unclear	low risk	low risk	unclear
Liu, 2023 [40]	unclear	unclear	unclear	unclear	unclear	unclear	unclear	unclear	low risk	unclear
Abou, 2022 [21]	unclear	unclear	unclear	unclear	unclear	unclear	unclear	unclear	low risk	unclear

Gan, 2022 [41]	unclear	low risk	unclear	low risk	unclear	unclear	unclear	unclear	low risk	unclear
Han, 2022 [42]	unclear	low risk	unclear	low risk	unclear	unclear	unclear	unclear	low risk	unclear
Li, 2021 [24]	unclear	unclear	unclear	low risk	low risk	unclear	low risk	unclear	low risk	unclear
Anselmo, 2021 [25]	unclear	unclear	unclear	unclear	unclear	unclear	unclear	unclear	low risk	unclear
Martins-Marques, 2020 [27]	unclear	low risk	unclear	low risk	unclear	unclear	unclear	unclear	low risk	unclear
Zhang, 2020 [43]	unclear	low risk	unclear	unclear	unclear	unclear	unclear	unclear	low risk	unclear
Chen, 2020 [44]	unclear	low risk	unclear	unclear	unclear	unclear	unclear	unclear	low risk	unclear
Vaskova, 2020 [45]	low risk	low risk	low risk	low risk	low risk	unclear	low risk	unclear	low risk	unclear
Luo, 2020 [46]	unclear	low risk	unclear	unclear	low risk	unclear	low risk	unclear	low risk	unclear
Rodriguez, 2018 [47]	unclear	low risk	unclear	unclear	unclear	unclear	unclear	low risk	low risk	unclear
Wang, 2016 [48]	unclear	unclear	unclear	unclear	unclear	unclear	unclear	unclear	low risk	unclear
Pironti, 2015 [49]	unclear	unclear	unclear	unclear	unclear	unclear	unclear	unclear	low risk	Unclear
